# Synthetic 5-amino-6-D-ribitylaminouracil paired with inflammatory stimuli facilitates MAIT cell expansion *in vivo*

**DOI:** 10.3389/fimmu.2023.1109759

**Published:** 2023-08-31

**Authors:** Adam G. Nelson, Huimeng Wang, Phoebe M. Dewar, Eleanor M. Eddy, Songyi Li, Xin Yi Lim, Timothy Patton, Yuchen Zhou, Troi J. Pediongco, Lucy J. Meehan, Bronwyn S. Meehan, Jeffrey Y. W. Mak, David P. Fairlie, Andrew W. Stent, Lars Kjer-Nielsen, James McCluskey, Sidonia B. G. Eckle, Alexandra J. Corbett, Michael N. T. Souter, Zhenjun Chen

**Affiliations:** ^1^ Department of Microbiology and Immunology, The University of Melbourne at the Peter Doherty Institute for Infection and Immunity, Melbourne, VIC, Australia; ^2^ State Key Laboratory of Respiratory Disease, Guangzhou Institute of Respiratory Disease, Guangzhou Medical University, Guangzhou, China; ^3^ Centre for Innate Immunity and Infectious Diseases, Hudson Institute of Medical Research, Clayton, VIC, Australia; ^4^ Institute for Molecular Bioscience, The University of Queensland, Brisbane, QLD, Australia; ^5^ Gribbles Veterinary Pathology, Clayton, VIC, Australia

**Keywords:** MAIT cells, 5-amino-6-D-ribitylaminouracil (5-A-RU), MAIT cell boosting, mouse model, CpG, IL-23

## Abstract

**Introduction:**

Mucosal-associated invariant T (MAIT) cells are a population of innate-like T cells, which mediate host immunity to microbial infection by recognizing metabolite antigens derived from microbial riboflavin synthesis presented by the MHC-I-related protein 1 (MR1). Namely, the potent MAIT cell antigens, 5-(2-oxopropylideneamino)-6-D-ribitylaminouracil (5-OP-RU) and 5-(2-oxoethylideneamino)-6-D-ribitylaminouracil (5-OE-RU), form via the condensation of the riboflavin precursor 5-amino-6-D-ribitylaminouracil (5-A-RU) with the reactive carbonyl species (RCS) methylglyoxal (MG) and glyoxal (G), respectively. Although MAIT cells are abundant in humans, they are rare in mice, and increasing their abundance using expansion protocols with antigen and adjuvant has been shown to facilitate their study in mouse models of infection and disease.

**Methods:**

Here, we outline three methods to increase the abundance of MAIT cells in C57BL/6 mice using a combination of inflammatory stimuli, 5-A-RU and MG.

**Results:**

Our data demonstrate that the administration of synthetic 5-A-RU in combination with one of three different inflammatory stimuli is sufficient to increase the frequency and absolute numbers of MAIT cells in C57BL/6 mice. The resultant boosted MAIT cells are functional and can provide protection against a lethal infection of *Legionella longbeachae*.

**Conclusion:**

These results provide alternative methods for expanding MAIT cells with high doses of commercially available 5-A-RU (± MG) in the presence of various danger signals.

## Introduction

Mucosal-associated invariant T (MAIT) cells are a subset of unconventional T cells that are defined, in part, by their recognition of antigens that are derived from microbial riboflavin biosynthesis (1, 2). The riboflavin precursor molecule 5-amino-6-D-ribitylaminouracil (5-A-RU) undergoes a condensation reaction with the reactive carbonyl species (RCS) glyoxal (G) and methylglyoxal (MG), to produce the potent MAIT cell antigens 5-(2-oxoethylideneamino)-6-D-ribitylaminouracil (5-OE-RU) and 5-(2-oxopropylideneamino)-6-D-ribitylaminouracil (5-OP-RU) (2, 3). These MAIT cell antigens are presented via the major histocompatibility complex class I (MHC-I)-related protein 1 (MR1) for recognition by the MAIT cell T cell receptor (TCR) (2, 4, 5). Accordingly, MAIT cells are activated by a wide range of bacteria and fungi that possess the riboflavin biosynthesis pathway (4, 6–17).

Due to the relatively low abundance of MAIT cells in unaltered laboratory mouse strains (0.1% of circulating αβ T cells) the study of MAIT cells in the context of diseases *in vivo* in mice can be difficult. Therefore, for the effective study of MAIT cells in the context of disease *in vivo*, methods of increasing the level of MAIT cells have been developed (13–15, 17–21). Significant MAIT cell expansion in mice has been demonstrated previously by administration of synthetic 5-OP-RU in combination with various danger signals (such as TLR agonists or defined cytokines), or bacterial infection with riboflavin autotrophs (14, 15). These “boosting” methods provide an inflammatory environment that provides sufficient co-stimulation to activate and increase the MAIT cell frequency and abundance in the tissues from ~0.1-1% of total αβ T cells in naive mice to upwards of 10-50% at day 7 post boosting and ∼10% during convalescence (15, 17). This increase in MAIT cell number has enabled the characterization of MAIT cells in mouse models. Nonetheless, there are limitations to the current published MAIT cell boosting methods. For instance, while infection with *Salmonella* Typhimurium, *Legionella longbeachae* or *Francisella tularensis* has been shown to markedly increase the absolute numbers of MAIT cells within mice (13, 15, 17), these infections also skew the expanded MAIT cell populations to distinct functional phenotypes and are also associated with significant non-MAIT αβ T cell accumulation, thus complicating downstream data interpretation (19). Furthermore, it may take several weeks for the bacteria to be cleared from the host (15, 17) creating a risk of residual bacterial contamination when performing MAIT cell isolation. This limits the suitability of infection-induced MAIT cell boosting in some models. Sterile MAIT cell-boosting strategies have also been developed and may be preferred for targeted augmentation of MAIT cells while minimising off-target effects on other immune cells (13). These boosting methods rely on co-administration of danger signals to simulate an infection, producing a co-stimulatory environment, which, in combination with synthetic 5-OP-RU, robustly increases MAIT cell abundance and frequencies amongst αβ T cells (13, 19).

Synthetic 5-OP-RU (22) is not commercially available but can be produced from the conversion of 5-A-RU in a non-enzymatic manner (23). Therefore, we assessed whether an alternative boosting strategy involving the administration of commercially available synthetic *5-A-RU* ± MG to mice together with co-stimuli, provided in the form of riboflavin pathway deficient bacteria, TLR9 agonist (CpG combo), or IL-23-Ig encoding plasmid DNA, was sufficient to induce MAIT cell expansion *in vivo*. Our results demonstrate that 5-A-RU ± MG, in combination with each one of the co-stimuli represent robust MAIT cell boosting strategies in mice.

## Results

### Co-administration of synthetic 5-A-RU alone or with MG facilitates the expansion of MAIT cells in mice following infection with riboflavin-deficient bacteria

First, we tested the ability of 5-A-RU to facilitate MAIT cell accumulation using bacterial infection as a means of co-stimulation. *S*. Typhimurium strain HW501 has a deletion of the *ribD* and *ribH* genes (hereafter referred to as *S.* Typhimurium *ΔRibD/H*) preventing the production of 5-A-RU and subsequently riboflavin (15, 24, 25) and is, therefore, unable to produce known MAIT cell antigens (15). We reasoned that a sufficiently high concentration of synthetic 5-A-RU with or without MG in conjunction with *S.* Typhimurium *ΔRibD/H* infection would facilitate the production of MAIT cell antigens *in vivo* and restore MAIT cell accumulation to the levels observed with a riboflavin producing strain of *S.* Typhimurium, BRD509 (15).

**Figure 1:**
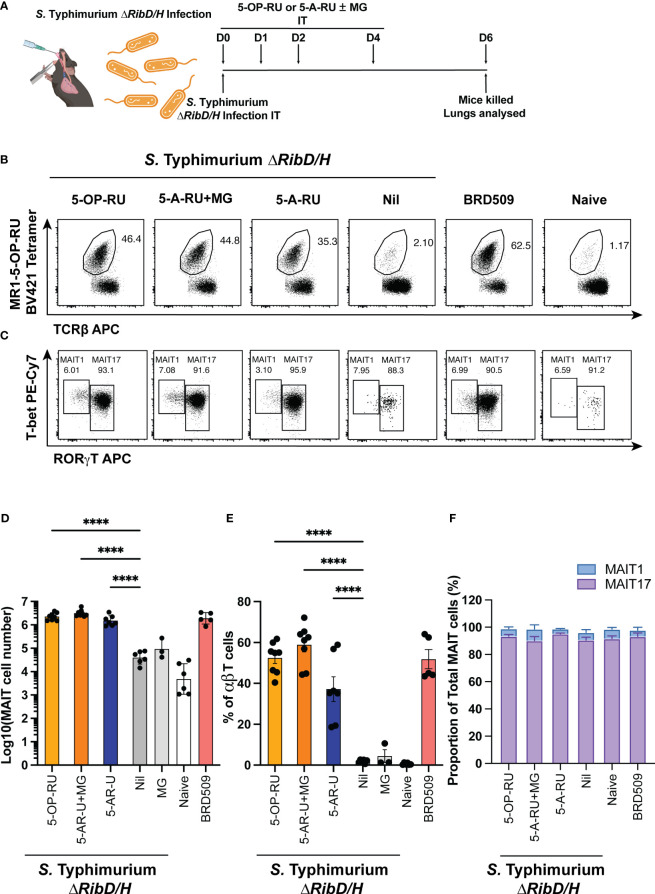
MAIT cell accumulation in mice infected with riboflavin pathway impaired bacteria (S. Typhimurium *ΔRibD/H*) supplemented with synthetic 5-OP-RU or 5-A-RU±MG. **(A)** Schematic outlining infection of mice with *S*. Typhimurium *∆RibD/H*, intratracheally (IT), and inoculation with four doses of either 5-OP-RU (50 pmol, 50 μL), 5-A-RU+MG (5-A-RU+MG: 32.5 nmol +110.5 nmol, 50 μL), 5-A-RU (5-A-RU: 32.5 nmol, 50 μL) or MG (110.5 nmol, 50 μL) on days 0 (D0), D1, D2 and D4, before harvest of lungs on D6. **(B)** Representative flow cytometry plots with gated MAIT cell frequency indicated and **(C)** representative flow cytometry plots with gated MAIT1 (T-bet high, RORγT low, left gate) and MAIT17 (T-bet high or low, RORγT high, right gate) frequencies. Bar graphs showing **(D)** absolute MAIT cell numbers, **(E)** MAIT cell frequency and **(F)** MAIT1 and MAIT17 as a proportion of total MAIT cells, from the lungs infected with 2 x 10^7^ CFU of S. Typhimurium ∆RibD/H and treated with four doses of either 5-OP-RU, 5-A-RU+MG 5-A-RU or MG IT on days 0, 1, 2 and 4; or infected with 2.5x10^6^ CFU *S*. Typhimurium BRD509 IT day 0, or naïve mice. Mice were killed and lungs were collected on day 6. Data show mean ± SEM and dots represent individual mice (n=3-8). Statistical significance is indicated by: **** (p<0.0001). One-way ANOVA with Tukey correction was performed on log-transformed data or percentage data. Data were pooled from two independent experiments.

Mice were infected intratracheally (IT) with *S*. Typhimurium *ΔRibD/H*, resuspended in PBS containing either 5-OP-RU, 5-A-RU, 5-A-RU that had been pre-incubated with MG (5-A-RU+MG), or MG alone. As a positive control, mice were infected with *S.* Typhimurium BRD509. Mice received three follow-up doses of either 5-OP-RU, 5-A-RU+MG, 5-A-RU, MG, or PBS on days 1, 2 and 4 (**Figure 1A**). Consistent with previous findings (15), both 5-OP-RU and 5-A-RU+MG fully restored MAIT cell frequency of αβ T cells and absolute numbers in the lungs of mice infected with *S.* Typhimurium *ΔRibD/H* to levels comparable to those in mice infected with *S.* Typhimurium BRD509 (**Figures 1B, D, E**) (15). Compared to mice that had received *S.* Typhimurium Δ*RibD/H* alone, mice that also received 5-A-RU displayed an ~36-fold increase in MAIT cell absolute numbers (**Figure 1D**). No significant change in total αβ T cell numbers or non-MAIT αβ T cell numbers was observed between treated groups, indicating that the 5-OP-RU, 5-A-RU+MG and 5-A-RU were acting specifically on MAIT cells (**Supplementary Figure 1A**). Similarly, no significant differences in other immune cell subsets (NKT cells, γδ T cells, NK cells, neutrophils, inflammatory monocytes, macrophages, conventional dendritic cells (cDCs, cDC1s and cDC2s) were detected among the groups of mice treated with the various compounds (**Supplementary Figure 2A**). The resultant expanded MAIT cells were predominantly CD4 and CD8 double negative across all treatments (**Supplementary Figure 3A**), in line with previous observations (15). Consistent with our previous work (14, 17, 19), infection with *S*. Typhimurium BRD509 induced a MAIT17 population, as defined by the expression pattern of transcription factors (TF) T-bet and RORγT (**Figures 1C, F**) (15, 19). Similarly, mice infected with *S*. Typhimurium *ΔRibD/H* with and without 5-OP-RU, 5-A-RU+MG and 5-A-RU administration followed the same MAIT17 phenotype suggesting a dominant IL-17 cytokine secreting capacity upon boosting (**Figures 1C, F**).

The accumulation of MAIT cells in the 5-A-RU alone group was ~2-fold lower than in the 5-A-RU+MG group (**Figure 1D**), suggesting that 5-A-RU alone *in vivo* is less efficient in inducing MAIT cell accumulation than 5-A-RU that had been pre-incubated with MG *in vitro*, which fully restored MAIT cell accumulation, relative to the *S.* Typhimurium *ΔRibD/H* with 5-OP-RU and *S.* Typhimurium BRD509 infections. Nevertheless, these data demonstrate that synthetic 5-A-RU is capable of stimulating MAIT cell accumulation *in vivo* when paired with a bacterial infection.

### TLR9 agonist and synthetic 5-A-RU ± MG stimulation induces robust MAIT cell accumulation in mice in an MR1 dependent manner

Next, we examined MAIT cell accumulation in response to 5-A-RU paired with the synthetic TLR9-agonists of B-class and P-class CpG (CpG combo) which elicits an inflammatory environment that activates a suite of immune cells and provides the necessary co-stimulatory signals for MAIT cell activation in mice together with 5-OP-RU (13, 26–29). We hypothesized that the combination of 5-A-RU, with or without MG, and CpG combo in the absence of pathogenic burden could stimulate MAIT cell accumulation.

**Figure 2:**
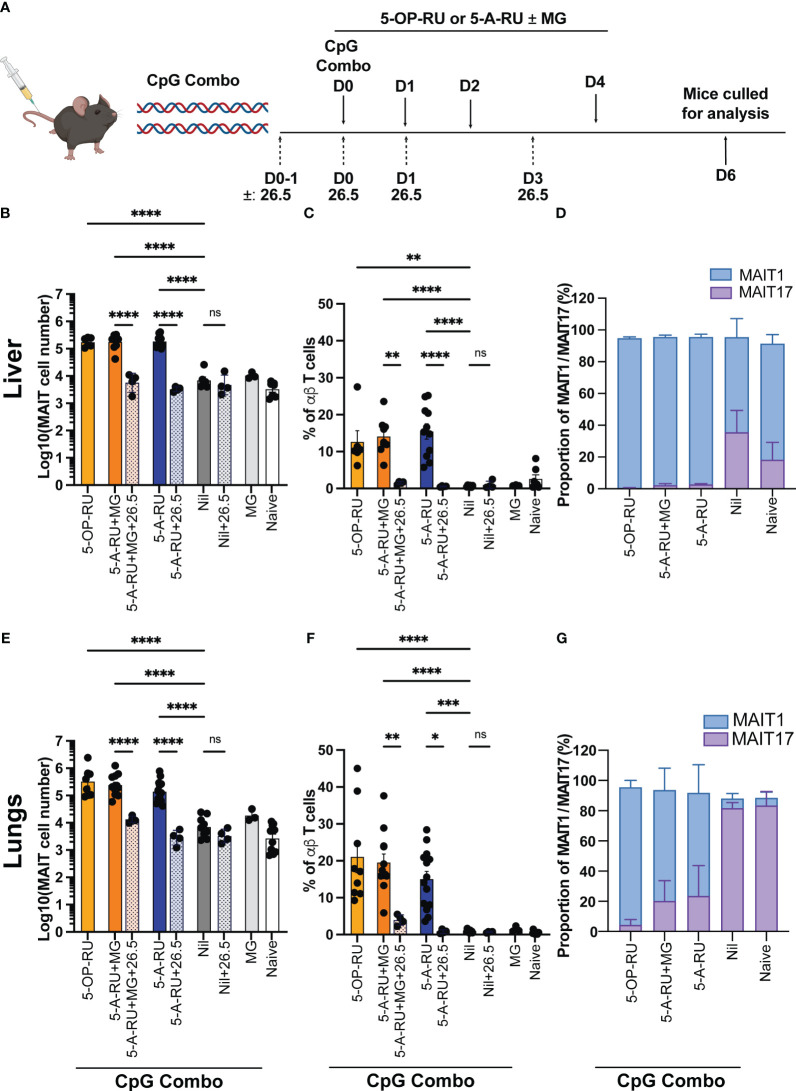
MAIT cell accumulation in mice inoculated with TLR-9 agonist CpG combo and synthetic 5-OP-RU or 5-A-RU ± MG in an MR1-dependent manner. **(A)** Schematic outlining CpG combo vaccination strategy. Mice were intravenously (IV) administered CpG and either 5-OP-RU, 5-A-RU+MG, or 5-A-RU on D0, D1, D2 and D4. Anti-MR1 monoclonal antibody 26.5 was administered on D0-1, D0, D1 and D3. Mice were killed on D6 and organs harvested for analysis. Bar graphs showing absolute MAIT cell numbers, MAIT cell percentage of αβ T cell and MAIT1 and MAIT17 as a proportion of total MAIT cells for the: liver **(B-D)**, or the lungs **(E-G)** of mice inoculated with 10 nmol of CpG combo and four doses of either 5-OP-RU (2 nmol, 200 μL), 5-A-RU+MG (5-A-RU+MG: 1.3 μmol +4.42 μmol, 200 μL), 5-A-RU (5-A-RU 1.3 μmol, 200 μL), MG (4.42 μmol, 200 μL) or PBS (200 μL) IV days 1, 2, 3, and 5 ± 4 doses of MR1 blocking monoclonal antibody 26.5 (250 mg, 200 μL). Data show mean ± SEM and dots represent individual mice (n=3-14). Statistical significance is indicated by ns (≥0.05) * (p<0.05), ** (p<0.01), *** (p<0.001), **** (p<0.0001). One-way ANOVA with Tukey correction was performed on log-transformed data or percentage data. Data were pooled from four independent experiments.

Mice were administered intravenously (IV) with CpG combo in combination with either 5-OP-RU, 5-A-RU pre-incubated with MG (5-A-RU+MG), 5-A-RU alone, MG alone, or PBS alone as a vehicle control (Nil), followed by additional doses of respective ligands on days 1, 2 and 5 (**Figure 2A**).

As expected, the 5-OP-RU and 5-A-RU+MG treated mice showed a robust increase in MAIT cell accumulation (30-60-fold in the lungs and 20-fold in the liver) when compared to control groups of mice treated with CpG and MG or CpG alone (**Figures 2B, C, E, F** and **Supplementary Figure 4**). Strikingly, in the livers of the mice treated with 5-A-RU, there was a similarly high increase in the accumulation of MAIT cells compared to the control groups, comparable to the mice treated with 5-OP-RU and 5-A-RU+MG (**Figures 2B, C**). This did not repeat in the lungs, where treatment of mice with 5-A-RU produced a 20-fold increase in MAIT cells from the control groups, compared to a 30-60-fold increase with 5-OP-RU or 5-A-RU+MG (**Figures 2E, F**). Consistent with this result, the percentage of MAIT cells of total αβ T cells closely reflected trends seen in the absolute number of MAIT cells generated by 5-OP-RU, 5-A-RU+MG and 5-A-RU. To confirm the robust expansion of MAIT cells exhibited by the boosting methods was MR1-dependent, we included control groups that received multiple doses of intraperitoneal anti-MR1 blocking antibodies (30) in addition to 5-A-RU+MG, 5-A-RU or CpG alone stimulation (**Figures 2B, C, E, F**). Functional blocking of MR1 significantly ameliorated MAIT cell accumulation in both lungs and liver in comparison to the non-blocked groups and are comparable in number to the Nil (CpG only) group and naive controls (**Figures 2B, C, E, F**). This confirmed the antigen-MR1 specific nature of the MAIT cell-boosting strategy.

Consistent with data using infection with *S.* Typhimurium *ΔRibD/H* to provide co-stimuli, we saw no significant differences in the accumulation of non-MAIT αβ T cells, or other immune cells between groups, demonstrating minimal off-target effects from these ligands (**Supplementary Figures 1B**, **2B**). Notably, we observed some tissue pathology (noticeable necrosis and/or thrombosis, **Supplementary Figure 5**) and leukocyte infiltration upon boosting with CpG combo in both the liver and the kidneys. However, tissue pathology was consistent between all treatment groups and the Nil group (CpG only). This suggests that the inflammation and leukocyte infiltration were likely due to the administration of the CpG combo and not the MAIT cell activating compounds 5-OP-RU, 5-A-RU+MG or 5-A-RU (**Supplementary Figure 5**). In contrast to infection with *S.* Typhimurium *ΔRibD/H*, CpG combo treatment together with 5-OP-RU or 5-A-RU+MG, yielded distinct coreceptor expression patterns by MAIT cells in both organs, involving a higher proportion of CD8^+^ MAIT cells compared to the 5-A-RU treatment groups or naïve mice (**Supplementary Figure 3B**). Despite these differences, expanded MAIT cells in both lungs and livers of mice stimulated with 5-A-RU+MG or 5-A-RU displayed a similar TF expression pattern as mice stimulated with 5-OP-RU (13, 19), indicating that in all three boosting regimens caused a skewing towards MAIT1 phenotype (**Figures 2D, G** and **Supplementary Figure 6A**). This phenotype was most prevalent in the liver (**Figure 2D** and **Supplementary Figure 6A**) but was also present within the lungs (**Figure 2G**, **Supplementary Figure 6A**). Together, these data demonstrate that 5-A-RU administered with a CpG combo can facilitate MAIT cell accumulation *in vivo* to a number and phenotype comparable to MAIT cells boosted with CpG combo and 5-OP-RU.

### Induction of IL-23 with administration of synthetic 5-A-RU induces MAIT cell accumulation in mice in an MR1 dependent manner

We have previously shown that MAIT cell accumulation can be achieved *in vivo* by inducing IL-23 expression in the presence of 5-OP-RU using hydrodynamic injection (HDI) of a DNA plasmid encoding IL-23-Ig (14, 19). This treatment provides a single defined co-stimulatory signal (IL-23) to the mice. Therefore, we tested if the combination of 5-A-RU and HDI of IL-23-Ig, in the absence of infection or other co-stimuli, could induce MAIT cell accumulation *in vivo*.

**Figure 3:**
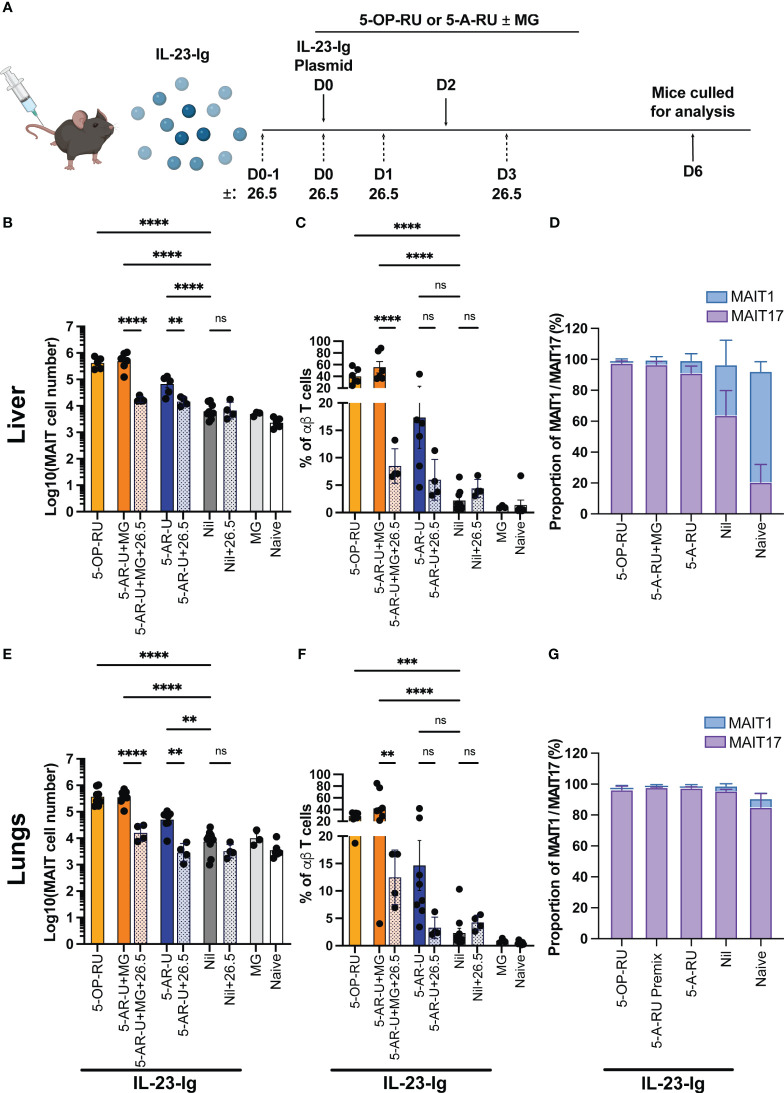
MAIT cell accumulation in mice inoculated with IL-23-Ig plasmid and synthetic 5-OP-RU or 5-A-RU±MG in an MR1-dependent manner **(A)** Schematic outlining IL-23-Ig vaccination strategy. Mice were administered IL-23-Ig plasmid DNA HDI and either 5-OP-RU, 5-A-RU+MG, or 5-A-RU D0, and D2, with or without MR1 blocking monoclonal antibody 26.5 administered D0-1, D0, D1 and D3. Mice were killed on D6 and organs harvested for analysis. Bar graphs showing absolute MAIT cell numbers, MAIT cell percentage of αβ T cell and MAIT1 and MAIT17 as a proportion of total MAIT cells for the: liver **(B–D)**, or the lungs **(E–G)** of mice inoculated with two doses of either 5-OP-RU (200 pmol, 200 μL), 5-A-RU+MG (5-A-RU+MG: 130 nmol +442 nmol, 200 μL), 5-A-RU (5-A-RU 130 nmol, 200 μL), MG (442 nmol, 200 μL) or PBS (200 μL), IV days 0 and 2. Data show mean ± SEM and dots represent individual mice (n=3-6). Statistical significance is indicated by ns (≥0.05), ** (p<0.01), *** (p<0.001); **** (p<0.0001). One-way ANOVA with Tukey correction was performed on log-transformed data or percentage data. Mann Whitney U tests were performed between 5-A-RU and 5-A-RU+26.5 as well as 5-A-RU and nil groups in lung absolute number panel E. Data were pooled from three independent experiments.

Mice were initially administered with IL-23-Ig plasmid DNA (HDI), followed by 5-OP-RU, 5-A-RU+MG, 5-A-RU, MG, or PBS (Nil) after eight hours and again on day 2 (**Figure 3A**). Consistent with previously published data (14), both 5-OP-RU and 5-A-RU+MG facilitated a robust, nearly 60 and 80-fold increase in liver MAIT cells, respectively, and an ~45 and ~39-fold increase in lung MAIT cells, respectively, when compared to the control IL-23 alone group (**Figures 3B, E** and **Supplementary Figure 7**). Surprisingly, MAIT cell accumulation in the 5-A-RU treated mice was limited to a 6-fold increase from the IL-23 group in the lungs and an 11-fold increase in the liver (**Figures 3B, E**). Similar to boosting with CpG combo, MAIT cell accumulation in both the lungs and liver in the presence of 5-A-RU+MG or 5-A-RU was inhibited by the addition of anti-MR1 blocking antibodies (**Figures 3B, C, E, F**). The expression of CD4 and CD8 by MAIT cells treated with IL-23 was consistent with previously published data (19), with the majority of MAIT cells being double negative for coreceptors, and minor populations of CD4 and CD8 single positive cells present in all treatment groups (**Supplementary Figure 3C**). Consistent with CpG-treated mice, we did not observe differences in the recruitment of non-MAIT αβ T cells, total αβ T cells or other immune cells between groups. (**Supplementary Figures 1C**, **2C**). As expected, no gross tissue pathology was observed in any of the treated groups (**Supplementary Figure 5**), however mild tissue inflammation and infiltration of leukocytes were noticeable in the liver and to a lesser extent in the kidneys of mice boosted with IL-23-Ig, suggesting that none of the compounds elicited additional tissue stress (**Supplementary Figure 5**).

A majority of expanded MAIT cells in both lungs and livers of mice stimulated with 5-A-RU+MG or 5-A-RU were MAIT17 based on their TF expression pattern (**Figures 3D, G** and **Supplementary Figure 6B**), consistent with MAIT cells expanded in mice stimulated with 5-OP-RU (13, 19).

Collectively these data show that the administration of high doses of the MAIT cell antigen precursor, 5-A-RU, with or without the addition of MG, can promote MAIT cell accumulation similarly to 5-OP-RU when accompanied by inflammatory stimuli.

### MAIT cells boosted with IL-23-Ig provide protective immunity to microbial challenge

**Figure 4:**
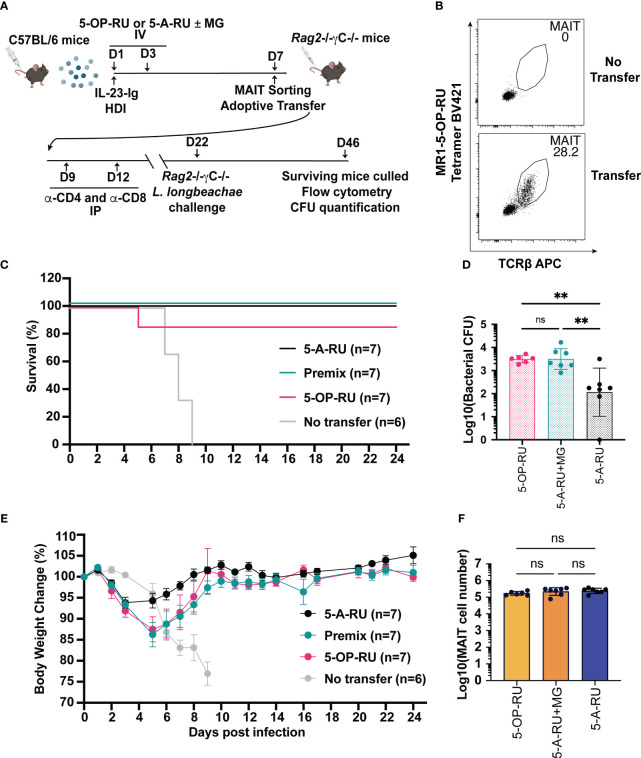
MAIT cells derived from IL-23-Ig vaccination scheme protect Rag2^-/-^γc^-/-^ mice from lethal challenge with *L. longbeachae*. **(A)** Schematic outlining vaccination schedule (all mice received 2 μg IL-23-Ig plasmid DNA via HDI and were inoculated with two doses of either 5-OP-RU (200 pmol, 200 μL), 5-A-RU+MG (5-A-RU+MG: 130 nmol +442 nmol, 200 μL) or 5-A-RU (5-A-RU 130 nmol, 200 μL)), adoptive transfer, conventional T cell depletion, and challenge with *L. longbeachae*. **(B)** Representative plots showing the MAIT cell profile of Rag2^-/-^γc^-/-^ mice transferred with MAIT cells compared to a Rag2-/-γc-/- mouse with no transfer. **(C)** Survival curve and **(D)** Weight change (%) of Rag2^-/-^γc^-/-^ mice transferred with 5-OP-RU, 5-A-RU+MG premix, 5-A-RU or without transfer, post-infection with *L. longbeachae*. **(E)** CFU of *L. longbeachae* and **(F)** MAIT cell numbers in the lungs of mice who survived bacterial challenge at day 24 post-infection. Data show mean ± SEM and dots represent individual mice (n=6-7). Statistical significance is indicated by ns (≥0.05) and ** (p<0.01). One-way ANOVA with Tukey correction was performed on log-transformed data or percentage data.

Boosted MAIT cells can be protective during microbial infection while also exhibiting the capacity to modulate other immune cells in various mouse models of disease (13, 15, 17, 20, 21). To establish whether cells boosted using IL-23-Ig and MAIT cell boosting compounds is sufficient to provide protective immunity, we used a model where MAIT cells from boosted C57BL/6 mice were transferred into immunocompromised (*Rag2* ^-/-^ *γC* ^-/-^) recipient mice before microbial challenge (13, 17). We chose to focus on IL-23-Ig boosted MAIT cells as this method provides the simplest adjuvant signal and microbial protection using CpG combo boosted MAIT cells has already been demonstrated (31). For challenge, we chose to use a lethal dose of the clinically relevant pathogen *L. longbeachae*, which has been extensively characterised previously using this model (17). MAIT cells from naïve C57BL/6 mice were boosted with IL-23-Ig HDI and 5-OP-RU, 5-A-RU+MG or 5-A-RU alone (**Figure 4A**) and sorted at day 6 as per the gating strategy in **Supplementary Figure 8**. The protective effect of the transferred boosted MAIT cells was evaluated by monitoring the survival following infection and the bacterial burden in the lungs of mice that survived to the experimental endpoint (**Figure 4A**). Efficiency of MAIT cell transfer was confirmed at the experimental endpoint (**Figure 4B**).

All mice without transferred MAIT cells succumbed to *L. longbeachae* infection by day 9 (**Figure 4C**), presumably by the uncontrolled growth of bacteria (17). By contrast, all but one of the mice with transferred MAIT cells from donor mice boosted with either 5-OP-RU, 5-A-RU+MG or 5-A-RU alone survived to the experimental endpoint (**Figure 4C**). All surviving mice recovered from the initial weight loss after infection to regain normal body conditions (**Figure 4E**). Interestingly, mice that had received transfer of 5-A-RU boosted MAIT cells lost the least weight and recovered earliest after infection (**Figure 4E**). The purity of transferred MAIT cells at the experimental endpoint (**Figure 4B**), confirmed the observed protection was solely mediated by the transferred MAIT cells, with no detectable contaminating non-MAIT αβ T cells (**Figure 4F**). Although the surviving mice had fully recovered or exceeded their baseline weight, surprisingly, bacterial counts remained substantial in the lungs 24 days post-infection (**Figure 4D**). Further, to our surprise, and in line with the trend in weight recovery between treatment groups, mice that received 5-A-RU boosted MAIT cells had a lower bacterial load in the lungs at the experimental endpoint compared to the mice that had received transfer of 5-OP-RU or 5-A-RU+MG boosted MAIT cells, despite each group receiving equal numbers of transferred MAIT cells and having similar levels of MAIT cell accumulation in the lungs 24 days post-infection (**Figure 4E**). In summary, these findings reflect the potential for MAIT cells to provide acute protection against infection as well as demonstrate their limitations in providing sterile immunity in the absence of a complete immune system, as we have demonstrated previously (13, 17).

## Methods

### Study design

Our study aimed to determine if MAIT cell accumulation could be achieved in mice treated by commercially available compounds (5-A-RU±MG) with alternative co-stimuli from infection, administration of synthetic TLR9 agonists (CpG combo) or cytokine (IL-23-Ig). To this end we assessed the capacity of boosted MAIT cells to combat a clinically significant bacterial pathogen. We also sought to address concerns over the potential off-target impact of MAIT cell boosting regimens on tissues within the mice and in other immune cell subsets. Various animal mouse models and flow cytometry of mouse organs was used to enumerate MAIT cells, other αβ T cells and other immune cell subsets. Mouse group numbers were selected for sufficient statistical power with one-way ANOVA.

### Mice

Mice were bred and housed under specific pathogen free conditions in the Melbourne Bioresource Facility at the Peter Doherty Institute for Infection and Immunity. C57BL/6 (6-12 weeks of age) weeks of age and *Rag2* ^-/-^ *γC* ^-/-^ (6-17 weeks of age) mice were used in experiments with the approval of the University of Melbourne’s Animal Ethics Committee (10201, 1814616 and 23211). Mice were killed at either the humane endpoint or at the experimental endpoint, as per ethics approval. Investigators were not blinded for substance administration, monitoring for endpoint survival, or subsequent analyses.

### Choice of compound dosage

For each boosting method (involving infection, CpG or IL-23 mediated co-stimulation) we used our previously established optimal doses of 5-OP-RU (50 pmol for administration with *S.* Typhimurium *ΔRibD/H*, 2 μmol for CpG combo and 200 pmol for IL-23-Ig) (13–15). We based the relative doses of 5-A-RU and 5-OP-RU in each experiment on matched MAIT cell accumulation observed *in vivo*. Matched accumulation was shown to be at an approximate 5-A-RU:5-OP-RU ratio of 650:1, leading us to choose a dose 650 times that of 5-OP-RU. The optimal ratio of 5-A-RU : MG to produce 5-OP-RU is 1:3.4, established in cell and MR1-free optimisation assays previously in the lab (unpublished data). A matched dose of 5-A-RU ± MG was used for downstream experiments after initial titration (**Supplementary Figure 9**):

1. Together with 2 × 10^7^ CFU *S.* Typhimurium *ΔRibD/H* (IT, 50 μL): 5-OP-RU (50 pmol); 5-A-RU (32.5 nmol)+MG (110.5 nmol) and 5-A-RU (32.5 nmol), then 3 more times with individual compounds alone at days 1, 2 and 4 (**Figure 1A**).
2. Together with 10 nmol CpG Combo (IV, 200 μL): 5-OP-RU (2 nmol); 5-A-RU (1.3 μmol)+MG (4.42 μmol) and 5-A-RU (1.3 μmol), then 3 more times with individual compounds alone at days 1, 2 and 4 (**Figure 2A**).
3. 6 hours post 2 μg IL-23-Ig HDI (IV, 200 μL): 5-OP-RU (200 pmol); 5-A-RU (130 nmol)+MG (442 nmol) and 5-A-RU (130 nmol), then one more time with individual compounds alone at day 2 (**Figure 3A**).

To prepare the 5-A-RU+MG premix, 5-A-RU was incubated with MG at a ratio of 1: 3.4 for 1 hour in the dark at room temperature in PBS. Compounds or premix were made in a master mix and were aliquoted and stored at -80°C for future use.

### Bacterial strains and infection/inoculation of mice

*S.* Typhimurium BRD509 and *S.* Typhimurium HW501 (*S.* Typhimurium *ΔRibD/H*) inoculums were prepared as described previously (15, 24, 25). In brief, the bacteria were cultured statically overnight in Luria Bertani (LB) broth containing 50 μg/mL streptomycin for *S.* Typhimurium BRD509, 30 μg/mL kanamycin and 50 μg/mL streptomycin with supplementation of riboflavin 20 μg/mL, for *S.* Typhimurium *ΔRibD/H*. Bacteria were then reinoculated for a further 2-4 hours with further static culture and allowed to reach OD_600_ of 0.4-0.6. Bacteria inoculums were made with the estimation that 1 OD600 = 5 x 10^8^ CFU/mL. Once made, the inoculum was then prepared using PBS ± compound(s) were added to the bacteria at the indicated concentrations, for inoculation of mice. A sample of each inoculum was retained and plated onto Luria Agar with antibiotics and supplementation of riboflavin (for *S.* Typhimurium *ΔRibD/H*) for CFU verification.

Mice were infected intratracheally (IT) on day 1 with *S.* Typhimurium *ΔRibD/H* (2 × 10^7^ CFU) in 50 μL of PBS containing indicated compound(s). Mice received either: 5-OP-RU (50 pmol, 50 μL), 5-A-RU+MG premix (5-A-RU+MG: 32.5 nmol +110.5 nmol, 50 μL), 5-A-RU (5-A-RU 32.5 nmol, 50 μL), MG (110.5 nmol, 50 μL) or PBS (50 μL). Mice were given subsequent IT inoculations of compound(s) in 50 μL at days 1, 2 and 4. Mice were killed at day 6, and lungs were collected for flow cytometric analysis (**Figure 1A**). As *S.* Typhimurium BRD509 forms a persisting infection with a much lower dose (2 × 10^6^ CFU), different doses for *S.* Typhimurium *ΔRibD/H* and *S.* Typhimurium BRD509 were chosen to simulate similar sublethal infection; producing comparable CFU counts at day 6 post-infection (15).

*L. longbeachae* NSW150 was grown at 37 °C in buffered yeast extract broth supplemented with 30–50 μg/mL of streptomycin overnight shaking at 180 rpm. For the infecting inoculum, bacteria were re-inoculated in prewarmed media for a further 2-4 hours culture to reach a log growing phase (OD_600_ ∼0.4). With the estimation that 1 OD600 = 5 × 10^8^ CFU/mL, bacteria were washed and diluted in PBS supplemented with 2% BYE for intranasal (IN) delivery to mice to a concentration of 2 × 10^4^ CFU/mL. A 50 μL inoculum (1500 CFU per mouse) was instilled through the nose of the mouse while unconscious. A sample of inoculum was spread onto buffered charcoal yeast extract agar plates with streptomycin for verification of bacterial concentration by CFU counting.

### Bacterial counts in infected lungs

Bacterial burden in mice was determined by counting CFU obtained from plating homogenised lungs on BCYE agar plates containing 30 μg/mL streptomycin and colonies counted after 4 days at 37 °C under aerobic conditions.

### Constructs, compounds, immunogens, and MR1 tetramers

The IL-23-Ig construct was generously provided by Professor Burkhard Becher, Switzerland. Synthetic 5-A-RU was purchased from the Ferrier Research Institute, Wellington, New Zealand through Prof. Gavin Painter. Synthetic 5-OP-RU was prepared in house as described previously (3). MG was purchased from Sigma. MR1-5-OP-RU and MR1-6-FP monomers and tetramers were made in-house as described previously (2). B-class and P-class CpG (CpG combo) with the sequence: 5’T*C*G*T*C*G*T*T*T*T*G*T*C*G*T*T*T*T*G*T*C*G*T*TT*CG*T*CG*A*CG*A*T*CG*G*C*G*CG*C*G*C*C*G-3′ (*phosphorothioate linkage) non-methylated cytosine–guanosine oligonucleotides was purchased from Integrated DNA Technologies, Singapore.

### Immunogen inoculation, hydrodynamic injection, antigen delivery and MR1 blockade

MAIT bosting regimen and delivery timeline are depicted in **Figures 1A**, **2A** and **3A**. Briefly, CpG combo (10 nmol) and either 5-OP-RU, 5-A-RU+MG premix, 5-A-RU, MG, or PBS were combined and administered to mice intravenously on day 0. Mice received individual compounds alone three more times on days 1, 2 and 4. MR1 specific monoclonal antibody (clone 26.5) was injected intraperitoneally (IP) at a dose of 250 μg in 200 μL of PBS 4 times (at days 0-1, 0, 1 and 4).

IL-23-Ig was administered to mice as described previously (14). In brief, mice received an HDI of 2 μg of IL-23-Ig PEF-BOS plasmid in 1.6-1.8 mL (8-10% of body weight) of TransIT-EE Hydrodynamic Delivery Solution (MIR 5340, Mirus Bio LLC, Madison, WI, USA) injected over a period of 10 seconds. Six-eight hours post-HDI, mice were administered with compounds (5-OP-RU, 5-A-RU or 5-A-RU+MG) intravenously in a volume of 200 μL. Mice received individual compounds again on day 2. Mice were monitored closely and were killed day 6 post-inoculation. Anti-MR1 antibody blocking was performed as described above (days 0-1, 0, 1 and 3).

### Preparation of cells for flow cytometry

Mouse organs were harvested from mice killed by CO_2_ asphyxiation. Single-cell preparation and MR1-5-OP-RU-tetramer staining for flow cytometry analysis were performed as described previously (24). In brief, lungs were perfused through the heart using 10 mL of cold Roswell Park Memorial Institute medium (RPMI) and were finely chopped before digestion using 3 mg/mL Collagenase III (Worthington, Cat#LS004182) with 5 μg/mL DNAse in RPMI (Gibco, Cat#21870-076) with 2% foetal calf serum (FCS) for 90 minutes at 37° C. Digested lung tissue was passed through a 70 μm strainer, pelleted and resuspended in 5 mL of a tris ammonium chloride solution (0.14 M NH_4_Cl (Sigma, Cat#A9434), 0.017 mM Tris (pH7.5, Sigma, Cat#T1503), then adjusted pH to 7.2 with 2 M HCl) for 5 minutes to lyse red blood cells, before neutralization with PBS with 2% FCS. Livers were perfused through the hepatic portal vein using 10 mL of cold RPMI and were passed through a 70 μm strainer before the separation of lymphocytes using Percoll gradient centrifugation (37% layered on top of 70%). Lymphocytes were removed, pelleted and red blood cells lysed as needed, as described with lungs, before resuspension in PBS with 2% FCS. Kidneys were finely chopped and digested, as with lungs using collagenase for 90 minutes. Samples were then passed through a 70 μm filter before being separated using Percoll gradient centrifugation as with livers. Lymphocytes were removed pelleted and lysed as with lungs before resuspension in PBS with 2% FCS. Spleens were collected and passed through a 70 μm filter, pelleted and lysed as described with lungs before resuspension in PBS with 2% FCS.

### Flow cytometry

Antibodies against murine CD4 (clone GK1.5, #552051, APC-Cy7, 1:200), CD19 (clone 1D3, #551001, PerCP Cy5.5, 1:200), CD45.2 (clone 104, #553772, FITC, 1:200), TCRβ (clone H57-597, #553174 and #553172, APC and PE, 1:200), CD44 (clone IM7, #612799, BUV737, 1:200) CD11b (clone M1/70, Cat #557657, APC-Cy7, 1:200), Ly6C (clone AL-21, Cat #563011, BV605, 1:200), CD103 (clone M290, Cat #740238, BUV395, 1:200), Ly6G (clone 1A8, Cat #560601, PE-Cy7, 1:200) were purchased from BD Biosciences (Franklin Lakes, NJ). Antibodies against murine CD8 (clone 53-6.7, #12-0081-83, PE, 1:800), T-bet (clone 4810, #25-5825-82, PE-Cy7 1:200), RORγT (clone B2D, #12-6981-80, APC, 1:200), CD49b (clone DX5, #25-5971-81, PE-Cy7, 1:200) were purchased from Thermo Fisher Scientific, eBioscience (San Diego CA). F4/80 (clone BM8, Cat #123116, APC, 1:200), MHCII (clone M5/114, Cat #107631, BV421, 1:200), CD11c (clone N418, Cat #117308, PE, 1:200), NK1.1 (clone PK136, Cat #108731, BV421, 1:200) were purchased from BioLegend (San Diego, CA).

Blocking Ab (26.5: anti-human MR1 MoAb), were prepared in-house.

Cell surface marker staining: Prior to MR1-5-OP-RU-tetramer staining, cell suspensions were blocked for non-specific staining using unlabelled MR1-6-FP-tetramer and anti-Fc receptor antibody (2.4G2; in house) for 15 minutes at room temperature. Cells were then stained with antibody and tetramer cocktail in PBS with 2% FCS containing 7-AAD (1:500) for 30 minutes at room temperature in the dark. Cells were analysed on an LSR Fortessa X20 with UV upgrade (BD Biosciences).

Intranuclear marker staining: For intranuclear staining, cells were stained in Fixable Viability Dye e780 (eBiosciences, San Diego, CA) for 30 minutes on ice before surface staining as above. Intranuclear staining was conducted using the Transcription Factor Buffer set (eBiosciences), as per the manufacturer’s instructions.

Data acquisition and analysis: Data were acquired on a LSR Fortessa X20 with UV upgrade (BD Biosciences) and data acquisition and analysis were carried out using Diva and FlowJo version 10.9.0 (BD Biosciences) respectively. Gating strategies are shown in **Supplementary Figures 8** and **10**.

### MAIT cell sorting, adoptive transfer, and infection challenge

MAIT cells enriched by various vaccination regimens with IL-23 were FACS sorted as previously described (17). Briefly, on day 6 of MAIT cell boosting, mice were killed, single-cell suspensions were prepared and live CD3+CD45+MR1-5-OP-RU tetramer+ cells were sorted using BD FACS Aria III (BD Biosciences). Next, 1 × 10^5^ MAIT cells were injected into the tail veins of recipient mice which subsequently received 0.1 mg each of anti-CD4 (GK1.5) and anti-CD8 (53-6.7) intraperitoneally on days 9 and 12 (3 and 7 days post transfer to *Rag2-/-γC-/-* mice) to deplete residual conventional T cells. Mice were rested for 2 weeks post-cell transfer to allow expansion and settlement of the MAIT cell population prior to subsequent infection challenge. Mice were then infected with 1500 CFU of *L. longbeachae* IN and weighed daily and assessed for visual signs of clinical disease, including inactivity, ruffled fur, laboured breathing and hunched behaviour. Animals that had lost ≥20% of their original body weight and/or displayed symptoms of pneumonia (deemed to have reached a humane end point) were humanely euthanised. Remaining mice were humanely euthanised at the experiment end time point (Day 24), and organs harvested for analysis of MAIT cells and bacterial burden.

### Histopathology

Livers and kidneys were fixed in 10% neutral buffered formalin, embedded in paraffin; 5 μm sections were stained with Hematoxylin and Eosin and examined by a pathologist (blinded) under light microscopy for tissue histopathology and leukocyte cell infiltration. The histopathology scoring system used was for liver: 0 (Normal): Aggregates of extramedullary haematopoiesis within periportal regions, around central veins and occasionally within sinusoids. 1 (Mild): Small scattered infiltrates of leukocytes within hepatic parenchyma or periportal regions (<1 per 10X field). 2 (Moderate): Average 1-5 aggregates of leukocytes per 10X field. Infiltrates of leukocytes within up to 25% of lobules. 3 (Severe): Widespread foci of leukocytes, average >5 aggregates of leukocytes per 10X field (often also necrosis and/or thrombosis). For kidney: 0 (Normal): No significant lesions. 1 (Mild): Rare interstitial inflammatory infiltrates.

### Statistical analysis

Statistical tests were performed using GraphPad Prism (version 9.1 LaJolla, CA). Normal Distribution was determined using Shapiro-Wilk Normality tests. Normally distributed data were compared using one-way analysis of variance (ANOVA) tests. Data sets found not to be normally distributed were compared using Mann-Whitney U tests.

## Discussion

MAIT cells are present at a relatively low frequency within most laboratory mouse strains (32) limiting their analysis in mouse models. Increasing MAIT cell numbers in mice to levels comparable to those in humans is a valuable approach when studying the role of MAIT cells in mouse models of infection or disease, as well as performing phenotypic and functional studies *ex vivo*. Several mouse MAIT cell boosting strategies have been developed to enable the greater study of MAIT cells, leading to key findings, such as both the protective and detrimental functional capacity of MAIT cells in controlling bacterial infections (13, 14, 17, 18, 20, 33, 34). These boosting strategies typically rely on the infection of mice with bacterial pathogens, or vaccination with 5-OP-RU which is not commercially available, difficult to produce and highly unstable (3). Here, we demonstrate three effective MAIT cell boosting strategies using commercially available 5-A-RU, the precursor to the potent MAIT cell antigens 5-OP-RU and 5-OE-RU (2, 16). Notably, the amount of 5-A-RU we used to stimulate equivalent MAIT cell response was 2-3 orders of magnitude greater compared to 5-OP-RU, as previous research had shown that only 1% of 5-A-RU is converted to 5-OP-RU in aqueous media (3), suggesting 5-A-RU may have a poor conversion rate to known MAIT cell antigens *in vivo*. A potential consequence of using significantly higher quantities of 5-A-RU during boosting is the likely higher concentration of contaminants including unreacted 5-A-RU and other hypothetical adducts with RCS other than MG and G that may spontaneously form *in vivo* and should be considered within the context of downstream experiments.

Whether 5-A-RU is a physiological MR1 ligand that can drive MAIT cell activation has not been fully resolved. To our knowledge, there is no direct evidence that 5-A-RU has the capacity to bind to MR1. Notably, 5-A-RU is not predicted to form a Schiff base with MR1 (2, 4), which is a key feature of the potent MAIT cell antigens 5-OP-RU and 5-OE-RU (2, 25, 35). However, non-Schiff base-forming compounds are known to have the capacity to bind MR1 and some are weak agonists for MAIT cells (35, 36). *In vitro* studies have demonstrated that the addition of 5-A-RU alone to cultured cells can stimulate MAIT cells, albeit to a lesser extent than with equivalent amounts of 5-OP-RU (2, 37), which is consistent with the relative accumulation of MAIT cells using our 5-A-RU/5-OP-RU boosting *in vivo*. The ubiquitous presence of RCS, including MG and G, in cell cultures suggests that the observed MAIT cell activation results from the spontaneous conversion of 5-A-RU to adducts such as 5-OP-RU and 5-OE-RU. However, it is difficult to discern whether these are the only RCS that are capable of forming physiologically relevant adducts with 5-A-RU within the cell culture media.

In addition to appropriate antigen stimulation, MR1-dependent activation of MAIT cells in the periphery requires co-stimulation (13–15, 19). However, a study using germ-free mice demonstrated that co-stimulation is not required to recapitulate MAIT cell expansion during development (37, 38). Interestingly, Legoux et al., showed that administration of low doses of 5-OP-RU, but not equivalent molar amounts of 5-A-RU, could restore MAIT cell development within the thymus in germ-free mice (38), suggesting that host-derived metabolites could not react with 5-A-RU *in vivo* and contribute to MAIT cell activation/accumulation (37, 38). Importantly, that study used low, matched doses of 5-A-RU and 5-OP-RU, which would likely result in unequal concentrations of MAIT cell agonists *in vivo* due to the poor conversion efficiency of 5-A-RU to other adducts. Thus, further investigations into whether a high concentration of 5-A-RU may also support MAIT cell development should be considered.

Despite most laboratory mouse strains having a low frequency of MAIT cells, a mouse line, B6-MAIT^CAST^, with substantially increased frequencies of MAIT cells, was generated by crossing C57BL/6 mice with CAST/EiJ mice (39). B6-MAIT^CAST^ mice have been proven to be useful in studying the fundamental biology of MAIT cells, given access to the increased MAIT cell numbers, especially within tissues and in naïve status (18, 40–43). However, some animal disease models involve genetic backgrounds other than C57BL/6 and it is time-consuming and costly to breed mice to a different genetic background and maintain all control mouse lines (e.g. MR1^-/-^). More importantly, B6-MAIT^CAST^ MAIT cells do not perfectly reflect the MAIT cell phenotype present in wild-type C57BL/6 mice. For instance, B6-MAIT^CAST^ MAIT cells produce type 2 cytokines and do not express the full complement of chemokine receptors when compared to MAIT cells derived from wild-type mice (39, 44). Thus, the MAIT cell boosting strategies presented here provide an alternative to genetically augmented MAIT cells.

Within the MAIT cell populations, we observed differences in CD4 and CD8 coreceptor expression as well as MAIT cell phenotype distribution depending on the co-stimuli provided. Infection with *S.* Typhimurium *ΔRibD/H* and administration of IL-23 skewed the population towards CD4, CD8 double negative MAIT cells as well as an increase in MAIT17; whereas CpG combo yielded a larger population of CD8^+^ MAIT cells and a bias towards MAIT1. MAIT1 cells are more enriched in the liver of mice where the majority of MAIT cells express CD8, while MAIT17 cells are more enriched at mucosal barriers where MAIT cells have a greater bias towards being coreceptor double negative (19). While not directly investigated here, this finding warrants further investigation for a potential role of coreceptor expression and MAIT cell functional phenotype (45, 46).

While we have demonstrated MAIT cell phenotype and function, we have not assessed the exhaustion status and longevity of MAIT cells using these three methods of boosting. Assessment of exhaustion markers such as PD-1, PD-L1 and Tim-3 (47) and the long-term persistence of boosted MAIT cells within mice warrants further investigation.

The optimised doses of individual components, particularly 5-A-RU, in the MAIT cell boosting regimens presented here are well tolerated by the mice and do not exceed the maximum dosage or number of inoculations previously reported for 5-OP-RU (18). The use of live bacteria, CpG and cytokine as adjuvants, has also been well established (14, 15, 20, 27, 48, 49). Despite observing some signs of pathology in the liver and kidneys for each boosting method, the level of pathology with 5-OP-RU treated mice as well as with adjuvant treated (Nil) groups were similar, indicating that the use of 5-A-RU and 5-A-RU+MG are viable alternatives to 5-OP-RU in sterile and non-sterile boosting methods.

In summary, we demonstrate three alternative methods for boosting MAIT cells *in vivo* distinct from previously published methods. We demonstrate 5-A-RU±MG and various co-stimuli that are all commercially available and sufficient to induce significant accumulation of MAIT cells *in vivo.* Boosted MAIT cells display consistent functional phenotypes based on transcription factor and coreceptor expression (13, 14, 19). Critically, these boosted MAIT cells were demonstrated to be capable of conferring protection in an adoptive transfer infection model using *L. longbeachae*, a clinically relevant pathogen. Thus, we believe these MAIT cell boosting strategies will be helpful in expanding the breadth of research on MAIT cells in various mouse models of disease.

## Data availability statement

The raw data supporting the conclusions of this article will be made available by the authors, without undue reservation.

## Ethics statement

The animal study was reviewed and approved by University of Melbourne, Animal Ethics Committee.

## Author contributions

AN and ZC conceived and designed the study. AN, ZC, HW, TP, PD, EE, YZ, SL, XL, MS, and TJP performed the experiments. JMc, SE, HW, MS, TP, and AC provided guidance and feedback for experiments. BM, LM, JM, DF, LK-N, SE, and MS provided reagents. AN analysed the data and prepared the figures. AN, ZC, MS, SE, TP, and AC wrote and/or edited the manuscript. AS examined tissue histopathology and prepared **Supplementary Figure 10**. The manuscript and figures were reviewed by all authors before submission. All authors contributed to the article.

## Supplementary material

Supplementary Figure 1 (Related to **Figures 1**–**3**): Non-MAIT and Total αβ T from lungs and livers of mice treated with MAIT cell boosting regimens. Bar graphs showing absolute numbers of non-MAIT αβ T or total αβ T in the lungs of **(A)** mice infected as per **Figure 1** or naïve mice. Lungs (top panels) and liver (bottom panels) of **(B)** CpG combo inoculated mice as per **Figure 2** or naïve mice. Lungs (top panels) and liver (bottom panels) of **(C)** IL-23-Ig plasmid inoculated mice as per **Figure 3** or naïve mice. Data show mean ± SEM and dots represent individual mice (n=3-14). Statistical significance is indicated by ns (≥0.05) * (p<0.05), ** (p<0.01), *** (p<0.001); **** (p<0.0001). One-way ANOVA with Tukey correction was performed on log-transformed data.

Supplementary Figure 2 (Related to **Figures 1**–**3**, **Supplementary Figure 9**): Immune cell subsets are not significantly influenced by inflammatory treatments: Bar graphs showing absolute numbers of NKT cells (CD1d-αGalCer tetramer+, TCRβ positive), γδ T cells (TCRβ-, γδTCR+), NK cells (TCRβ-, NK1.1+, CD49b), Neutrophils (Ly6G+, CD11b+), Inflammatory monocytes (Ly6G-, Ly6C+, CD11b+), Macrophages (F4/80+, CD11c+), conventional dendritic cells (DCs) (F4/80-, CD11c+, MHCII+), migratory CD103+ conventional DC1 (F4/80-, CD11c+, MHCII+, CD103+) conventional DC2 (F4/80-, CD11c+, MHCII+, CD11b+). **(A)** Mice infected with *S.* Typhimurium *ΔRibD/H*, *S.* Typhimurium BRD509, or naïve mice as per **Figure 1**. Liver (top panels) and lungs (bottom panels) of **(B)** CpG combo inoculated mice, as per **Figure 2** or naïve mice. Liver (top panels) and lungs (bottom panels) of **(C)** IL-23-Ig plasmid inoculated mice, as per **Figure 3** or naïve mice. Of note in the livers of mice treated with **(B)** CpG and compounds or **(C)** IL-23-Ig and compounds, we were unable to detect macrophages as distinguished by ND. Data show mean ± SEM and dots represent individual mice (n=3-14). Statistical significance is indicated by ns (≥0.05), * (p<0.05), ** (p<0.01). One-way ANOVA with Tukey correction was performed on log-transformed data or percentage data.

Supplementary Figure 3 (Related to **Figures 1**–**3**): Coreceptor (CD4 & CD8) usage by MAIT cells, non-MAIT αβ T and total αβ T for MAIT cell boosting regimens. Bar graphs showing the breakdown of CD4 and CD8 Coreceptor usage, CD4^+^ cells are in pink, CD8^+^ cells are in green, CD4 CD8 double-positive cells are in grey and CD4 CD8 double-negative cells are in white. **(A)** Coreceptor breakdown in the lungs of mice infected with *S.* Typhimurium *ΔRibD/H*, *S.* Typhimurium BRD509 infected or naïve mice as per **Figure 1** for MAIT cells (top panel), non-MAIT T cells (middle panel) and total T cells (bottom panel). **(B)** Coreceptor breakdown of CpG combo inoculated as per **Figure 2** or naïve mice, for liver (left) and lungs (right) across MAIT cells (top panels), non-MAIT T cells (middle panels) and T cells total (bottom panels). **(C)** Coreceptor breakdown IL-23-Ig plasmid inoculated mice as per **Figure 3** or naïve mice, for liver (left) and lungs (right) across MAIT cells (top panels), non-MAIT T cells (middle panels) and T cells total (bottom panels). Coreceptor usage was gated as per **Supplementary Figure 1** and calculated as a proportion of the total cell number for each cell type. Data show mean ± SD (n=3-14).

Supplementary Figure 4 (Related to **Figure 2**): MAIT cell enumeration from boosting with CpG combo, with and without MAIT cell boosting compounds. **(A)**, Representative flow cytometry plots of MAIT cells from the liver, lungs, kidney, spleen, and blood of mice treated with CpG plus various compounds and controls. MAIT cells were gated and MAIT cell frequency as a percentage of total αβ T cells shown. Absolute number **(B-D)** and frequency **(E-G)** of MAIT cells as a percentage of all αβ T cells displayed from indicated groups of mice: Blood: **(B, E)**; Kidney: **(C, F)**; Spleen: **(D, G)**. Data show mean ± SEM and dots represent individual mice (n= 3). Statistical significance is indicated by ns (≥0.05) * (p<0.05), ** (p<0.01). One-way ANOVA with Tukey correction was performed on log-transformed data or percentage data.

Supplementary Figure 5 (Related to and **Figures 2**, **3**): Histopathology of liver and kidneys in MAIT cell boosting strategies: Histopathology of liver and kidneys in MAIT cell boosting strategies: Graphs showing the histopathological score in the livers **(G, H)** and kidneys **(I, J)** of mice treated with CpG combo **(G, I)** or IL-23 **(H, J)**. Coloured graph bars represent average of histopathology scores. Each dot represents one mouse (n=2-3). **(E-J)** Representative hematoxylin and eosin (H&E) staining images of liver and kidney samples as per histopathology grading scores (Bar=100 μm, bottom-right in each image): **(E)** Normal mouse liver with no inflammation present. **(F)** Mouse liver with grade 1 inflammatory lesions. Low numbers of infiltrates of mononuclear leukocytes (arrow) are present within the parenchyma. **(G)** Mouse liver with grade 2 inflammatory lesions. Moderate numbers of infiltrates of mononuclear leukocytes (arrows) are present within the parenchyma. **(H)** Mouse liver with grade 3 inflammatory lesions. Frequent infiltrates of mononuclear leukocytes (arrows) are present throughout the parenchyma, with a vascular thrombus (asterisk) also present. **(I)** Normal mouse kidney displaying no histological lesions. **(J)** Mouse kidney displaying mild focal interstitial infiltrates of mononuclear leukocytes (arrow).

Supplementary Figure 6 (Related to and **Figures 2**, **3**): MAIT cell functional phenotyping of MAIT1 and MAIT17 by expression of hallmark transcription factors RORγT and T-bet. Flow cytometry plots of MAIT cells from indicated organs expressing RORγT and T-bet are depicted from mice treated with CpG **(A)** and IL-23-Ig **(B)**, with or without MAIT stimulating compounds as indicated. MAIT1 (T-bet high, RORγT low, left gate) and MAIT17 (T-bet high or low, RORγT high, right gate) were shown in the gate. The numbers indicate the abundance as a percentage (%) of total MAIT cells.

Supplementary Figure 7 (Related to **Figure 3**): MAIT cell enumeration in various tissue from boosting with IL-23-Ig with and without MAIT cell boosting compounds. **(A**) Representative flow cytometry plots of MAIT cells from the liver, lungs, kidney, spleen, and blood of mice treated with IL-23-Ig plus various compounds and controls. MAIT cells were gated and MAIT cell frequency as a percentage of total αβ T cells shown. Absolute number **(B-D)** and frequency **(E-G)** of MAIT cells as a percentage of total αβ T were displayed from indicated groups of mice: Blood: **(B, E)**; Kidney: **(C, F)**; Spleen: **(D, G)**. Data show mean ± SEM and dots represent individual mice (n= 3). Statistical significance is indicated by ns (≥0.05) * (p<0.05), ** (p<0.01), *** (p<0.001); **** (p<0.0001). One-way ANOVA with Tukey correction was performed on log-transformed data or percentage data.

Supplementary Figure 8 (Related to all Figures, except **Supplementary Figure 5**): Cytometric gating strategy for murine MAIT cells. Murine MAIT cells were identified by gating on lymphocytes and then excluding doublets using forward and side scatter (height *vs.* area). 7-AAD^-^ CD45^+^ events (live lymphocytes) were gated, and αβ T were identified as TCRβ^+^CD19^-^. Auto-fluorescent cells were excluded using violet channel 525/50 (autofluorescence exclusion gate) and any residual non-T cells were excluded based on negative MR1-5-OP-RU tetramer staining (total T cells). Non-MAIT αβ T were gated as TCRβ^+^ MR1-5-OP-RU tetramer^-^, and MAIT cells were gated as MR1-5-OP-RU tetramer+ TCRβ^intermediate^ cells. Coreceptor expression of CD4 and CD8 was gated on non-MAIT, MAIT and total T cell populations. MAIT1 (T-bet high, RORγT low) and MAIT 17 (T-bet high or low, RORγT high) cells were separated based on T-bet and RORγT expression. This gating strategy was used for all experiments.

Supplementary Figure 9 Bar graphs showing absolute numbers of MAIT cells in the lungs (top panels) or livers (bottom panels) of **(A)** Mice infected with 2 x 10^7^ CFU of S. Typhimurium *∆RibD/H* and administered four doses (as in **Figure 1A**) of either 5-OP-RU (50 pmol, 50 μL), 5-A-RU+MG (5-A-RU+MG: 32.5 nmol +110.5 nmol, 50 μL), a titration of 5-A-RU (5-A-RU (H): 32.5 nmol, 50 μL, 5-A-RU (M): 13 nmol, 50 μL or 5-A-RU (L): 2.6 nmol, 50 μL) or MG (110.5 nmol, 50 μL) IT, or infected with 2.5 x 10^6^ CFU *S*. Typhimurium BRD509 IT, or naïve mice. **(B)** CpG combo inoculated mice with four doses (as in **Figure 2A**)  of either 5-OP-RU (2 nmol, 200 μL), 5-A-RU+MG (5-A-RU+MG: 1.3 μmol +4.42 μmol, 200 μL), a titration of 5-A-RU (5-A-RU (H): 1.3 μmol, 200 μL, 5-A-RU (M): 260 nmol, 200 μL or 5-A-RU (L): 52 nmol, 200 μL), MG (4.42 μmol, 200 μL) or PBS (200 μL) IV or naïve mice. **(C)** IL-23-Ig plasmid inoculated mice with two doses (as in **Figure 3A**) of either 5-OP-RU (200 pmol, 200 μL), 5-A-RU+MG premix (5-A-RU+MG: 130 nmol +442 nmol, 200 μL) IV, a four-dose titration of 5-A-RU (5-A-RU (H): 130 nmol, 200 μL, 5-A-RU (M): 26 nmol, 200 μL or 5-A-RU (L): 5.2 nmol, 200 μL), MG (442 nmol, 200 μL) or naïve mice. Data show mean ± SEM (where n ≥ 3) and dots represent individual mice (n=2, 3 and 5). Statistical analysis was performed on log-transformed data and was performed using a one-way ANOVA with multiple comparisons. *p<0.05. Experiments were performed twice with similar results.

Supplementary Figure 10 (Related to **Figures 1**–**3**, **Supplementary Figures 2**, **3**): Cytometric gating strategy for other non-MAIT immune cell subsets. Non-MAIT immune cell subsets were gated similarly as in **Supplementary Figure 1**. Live (7AAD-) single immune cells (CD45.2+) were gated out first, then various cell subsets are defined and enumerated with the following cell surface markers. NKT cells (CD1d-αGalCer tetramer+, TCRβ positive), γδ T cells (TCRβ-, γδTCR+), NK cells (TCRβ-, NK1.1+, CD49b), Neutrophils (Ly6G+, CD11b+), Inflammatory monocytes (Ly6G-, Ly6C+, CD11b+), Macrophages (F4/80+, CD11c+), total conventional dendritic cells (DCs) (F4/80-, CD11c+, MHCII+), conventional DC1 (F4/80-, CD11c+, MHCII+, CD103+) conventional cDC2 (F4/80-, CD11c+, MHCII+, CD11b+), were all identified based on the gating strategy above. This strategy was used for all experiments enumerating all non-MAIT and non-T cell subsets.
