# Supplementary figures and images for: Synthetic 5-amino-6-D-ribitylaminouracil paired with inflammatory stimuli facilitates MAIT cell expansion in vivo
Source: Front Immunol. 2023 Aug 31;14:1109759. doi: 10.3389/fimmu.2023.1109759 (PMC10500299; doi:10.3389/fimmu.2023.1109759)

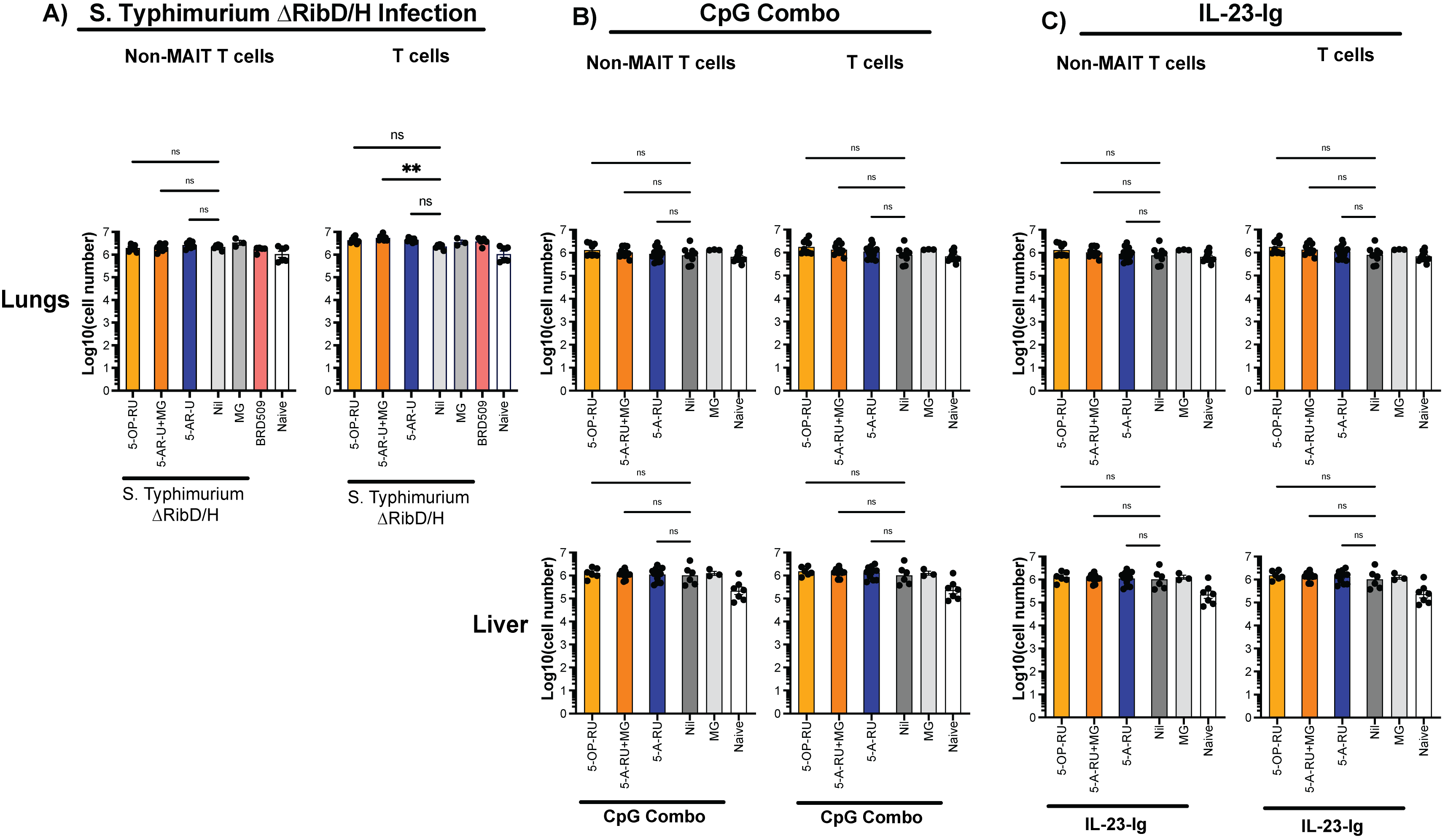

Supplement: Supplementary Figure 1 — (Related to Figures 1 – 3 ): Non-MAIT and Total αβ T from lungs and livers of mice treated with MAIT cell boosting regimens. Bar graphs showing absolute numbers of non-MAIT αβ T or total αβ T in the lungs of (A) mice infected as per Figure 1 or naïve mice. Lungs (top panels) and liver (bottom panels) of (B) CpG combo inoculated mice as per Figure 2 or naïve mice. Lungs (top panels) and liver (bottom panels) of (C) IL-23-Ig plasmid inoculated mice as per Figure 3 or naïve mice. Data show mean ± SEM and dots represent individual mice (n=3-14). Statistical significance is indicated by ns (≥0.05) * (p<0.05), ** (p<0.01), *** (p<0.001); **** (p<0.0001). One-way ANOVA with Tukey correction was performed on log-transformed data. [file Image_1.tif]

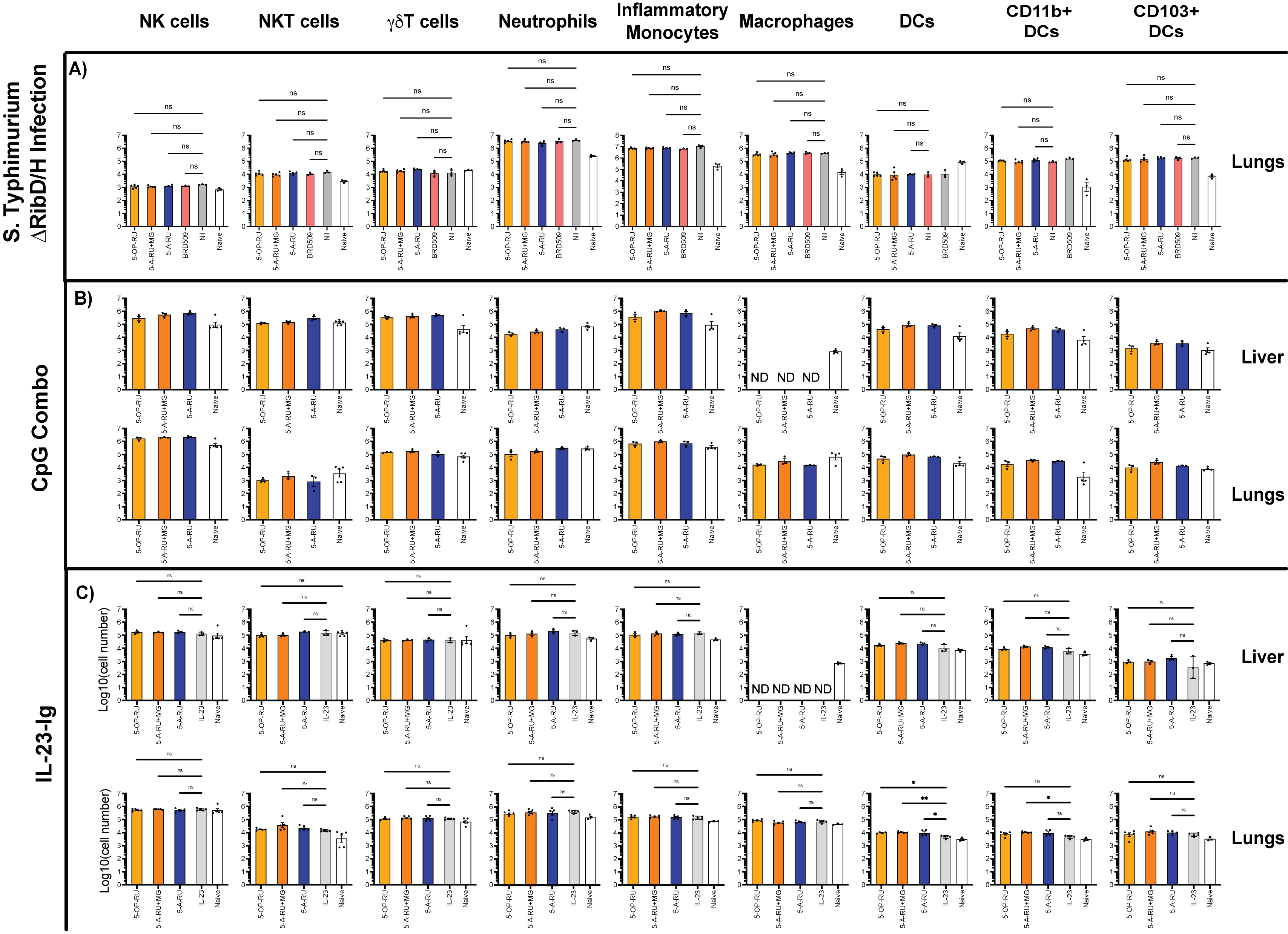

Supplement: Supplementary Figure 2 — (Related to Figures 1 – 3 , Supplementary Figure 9 ): Immune cell subsets are not significantly influenced by inflammatory treatments: Bar graphs showing absolute numbers of NKT cells (CD1d-αGalCer tetramer+, TCRβ positive), γδ T cells (TCRβ-, γδTCR+), NK cells (TCRβ-, NK1.1+, CD49b), Neutrophils (Ly6G+, CD11b+), Inflammatory monocytes (Ly6G-, Ly6C+, CD11b+), Macrophages (F4/80+, CD11c+), conventional dendritic cells (DCs) (F4/80-, CD11c+, MHCII+), migratory CD103+ conventional DC1 (F4/80-, CD11c+, MHCII+, CD103+) conventional DC2 (F4/80-, CD11c+, MHCII+, CD11b+). (A) Mice infected with S. Typhimurium ΔRibD/H, S. Typhimurium BRD509, or naïve mice as per Figure 1 . Liver (top panels) and lungs (bottom panels) of (B) CpG combo inoculated mice, as per Figure 2 or naïve mice. Liver (top panels) and lungs (bottom panels) of (C) IL-23-Ig plasmid inoculated mice, as per Figure 3 or naïve mice. Of note in the livers of mice treated with (B) CpG and compounds or (C) IL-23-Ig and compounds, we were unable to detect macrophages as distinguished by ND. Data show mean ± SEM and dots represent individual mice (n=3-14). Statistical significance is indicated by ns (≥0.05), * (p<0.05), ** (p<0.01). One-way ANOVA with Tukey correction was performed on log-transformed data or percentage data. [file Image_2.tif]

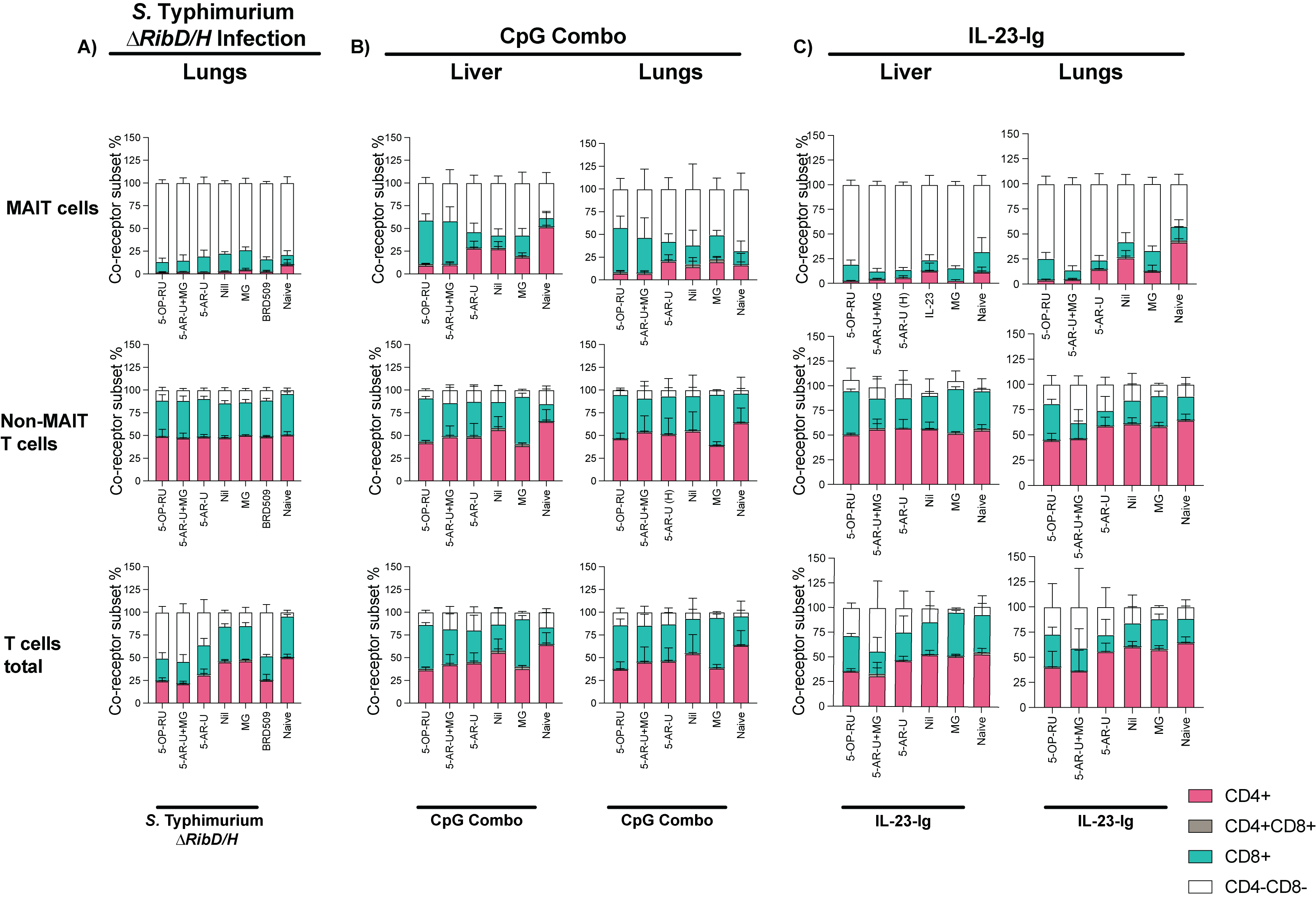

Supplement: Supplementary Figure 3 — (Related to Figures 1 – 3 ): Coreceptor (CD4 & CD8) usage by MAIT cells, non-MAIT αβ T and total αβ T for MAIT cell boosting regimens. Bar graphs showing the breakdown of CD4 and CD8 Coreceptor usage, CD4+ cells are in pink, CD8+ cells are in green, CD4 CD8 double-positive cells are in grey and CD4 CD8 double-negative cells are in white. (A) Coreceptor breakdown in the lungs of mice infected with S. Typhimurium ΔRibD/H, S. Typhimurium BRD509 infected or naïve mice as per Figure 1 for MAIT cells (top panel), non-MAIT T cells (middle panel) and total T cells (bottom panel). (B) Coreceptor breakdown of CpG combo inoculated as per Figure 2 or naïve mice, for liver (left) and lungs (right) across MAIT cells (top panels), non-MAIT T cells (middle panels) and T cells total (bottom panels). (C) Coreceptor breakdown IL-23-Ig plasmid inoculated mice as per Figure 3 or naïve mice, for liver (left) and lungs (right) across MAIT cells (top panels), non-MAIT T cells (middle panels) and T cells total (bottom panels). Coreceptor usage was gated as per Supplementary Figure 1 and calculated as a proportion of the total cell number for each cell type. Data show mean ± SD (n=3-14). [file Image_3.tif]

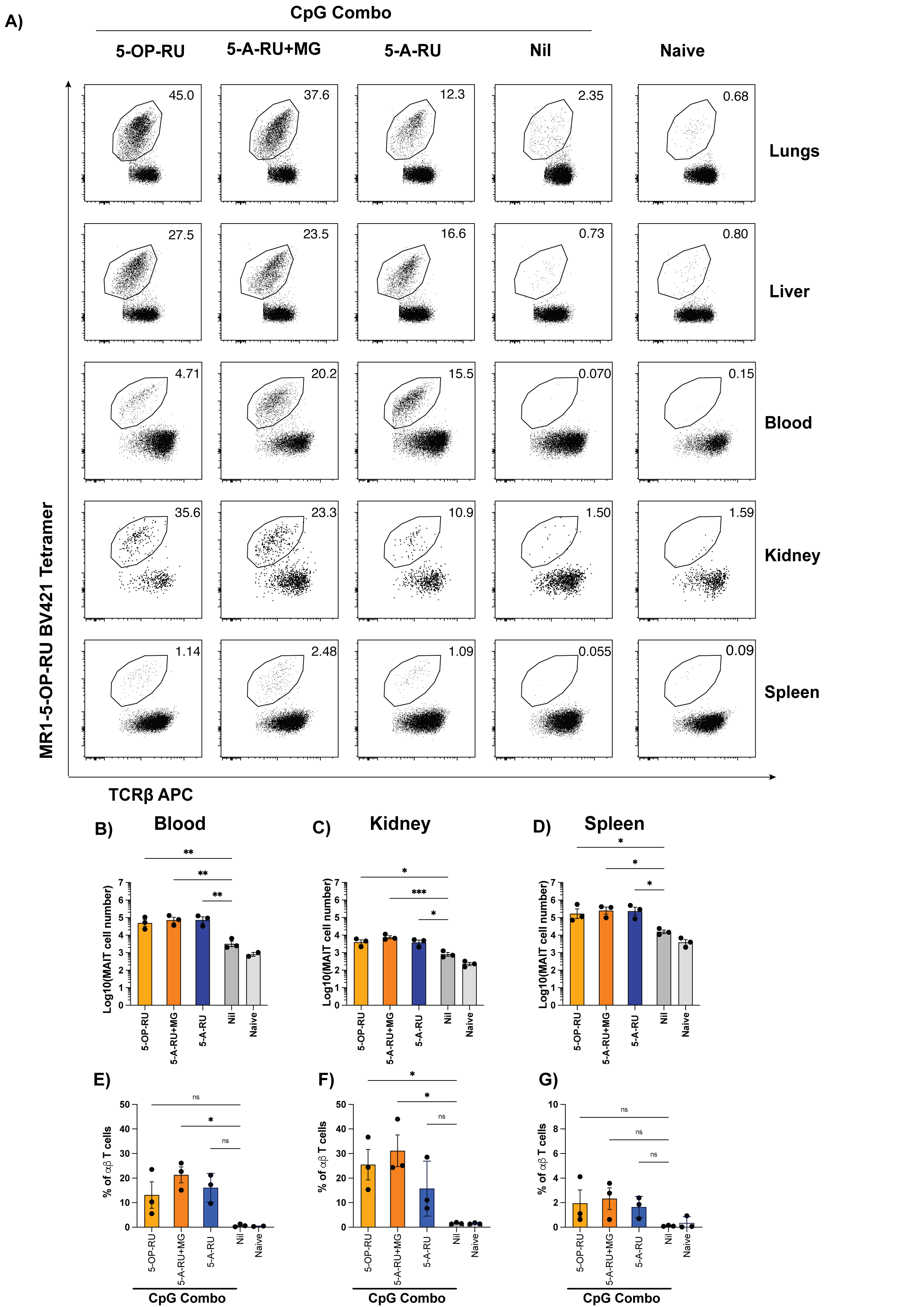

Supplement: Supplementary Figure 4 — (Related to Figure 2 ): MAIT cell enumeration from boosting with CpG combo, with and without MAIT cell boosting compounds. (A), Representative flow cytometry plots of MAIT cells from the liver, lungs, kidney, spleen, and blood of mice treated with CpG plus various compounds and controls. MAIT cells were gated and MAIT cell frequency as a percentage of total αβ T cells shown. Absolute number (B-D) and frequency (E-G) of MAIT cells as a percentage of all αβ T cells displayed from indicated groups of mice: Blood: (B, E); Kidney: (C, F); Spleen: (D, G). Data show mean ± SEM and dots represent individual mice (n= 3). Statistical significance is indicated by ns (≥0.05) * (p<0.05), ** (p<0.01). One-way ANOVA with Tukey correction was performed on log-transformed data or percentage data. [file Image_4.tif]

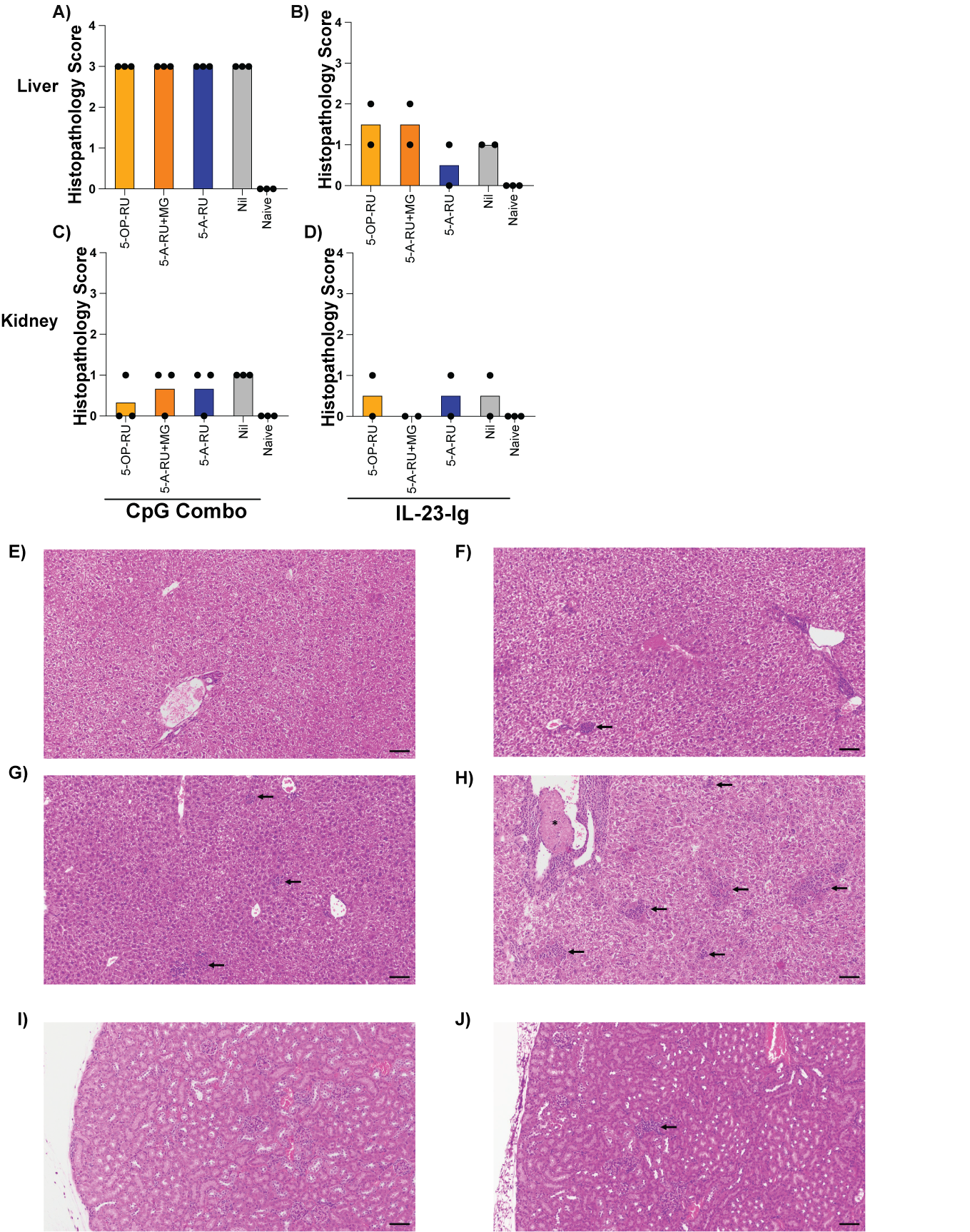

Supplement: Supplementary Figure 5 — (Related to and Figures 2 , 3 ): Histopathology of liver and kidneys in MAIT cell boosting strategies: Histopathology of liver and kidneys in MAIT cell boosting strategies: Graphs showing the histopathological score in the livers (G, H) and kidneys (I, J) of mice treated with CpG combo (G, I) or IL-23 (H, J). Coloured graph bars represent average of histopathology scores. Each dot represents one mouse (n=2-3). (E-J) Representative hematoxylin and eosin (H&E) staining images of liver and kidney samples as per histopathology grading scores (Bar=100 μm, bottom-right in each image): (E) Normal mouse liver with no inflammation present. (F) Mouse liver with grade 1 inflammatory lesions. Low numbers of infiltrates of mononuclear leukocytes (arrow) are present within the parenchyma. (G) Mouse liver with grade 2 inflammatory lesions. Moderate numbers of infiltrates of mononuclear leukocytes (arrows) are present within the parenchyma. (H) Mouse liver with grade 3 inflammatory lesions. Frequent infiltrates of mononuclear leukocytes (arrows) are present throughout the parenchyma, with a vascular thrombus (asterisk) also present. (I) Normal mouse kidney displaying no histological lesions. (J) Mouse kidney displaying mild focal interstitial infiltrates of mononuclear leukocytes (arrow). [file Image_5.tif]

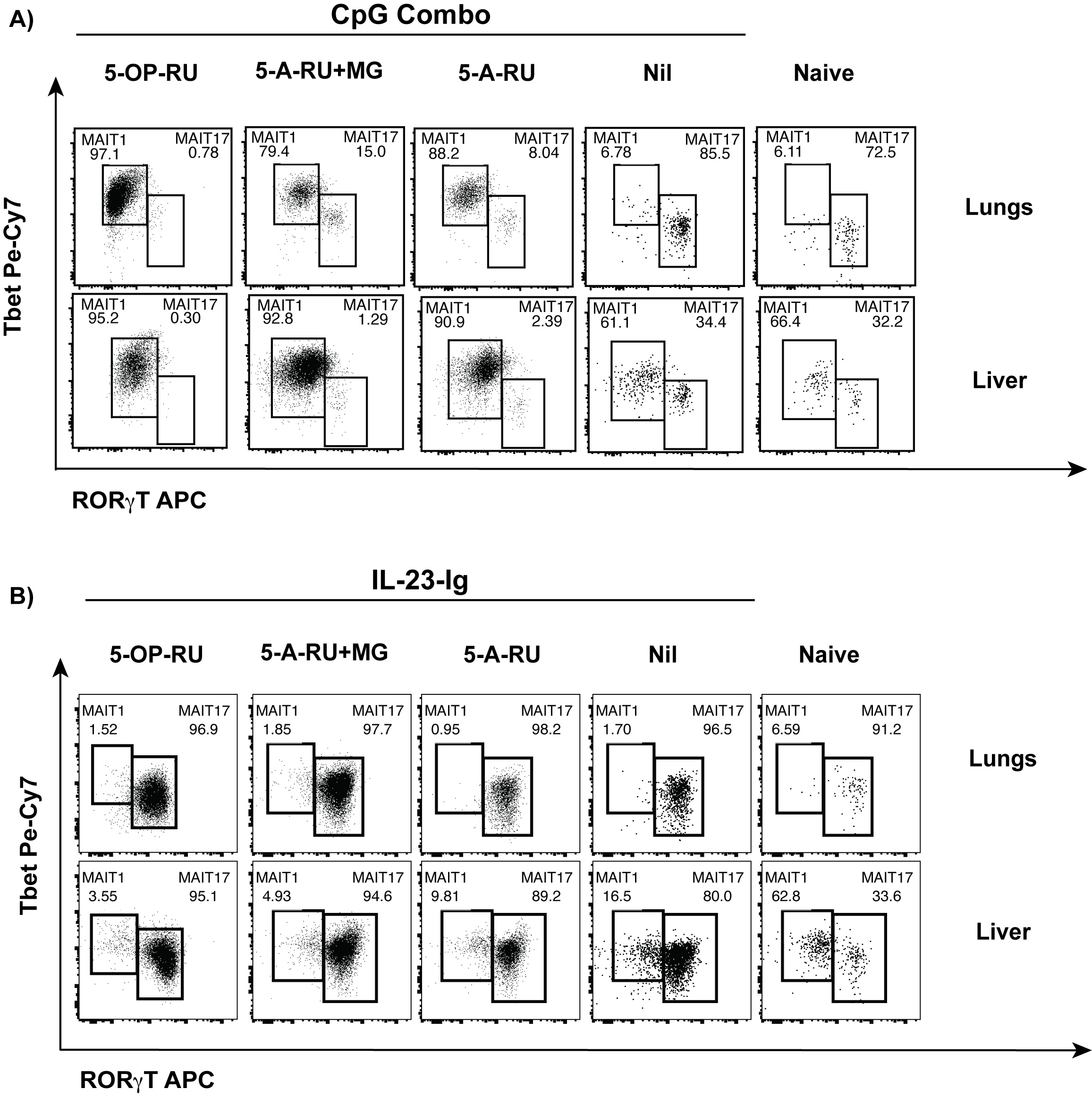

Supplement: Supplementary Figure 6 — (Related to and Figures 2 , 3 ): MAIT cell functional phenotyping of MAIT1 and MAIT17 by expression of hallmark transcription factors RORγT and T-bet. Flow cytometry plots of MAIT cells from indicated organs expressing RORγT and T-bet are depicted from mice treated with CpG (A) and IL-23-Ig (B), with or without MAIT stimulating compounds as indicated. MAIT1 (T-bet high, RORγT low, left gate) and MAIT17 (T-bet high or low, RORγT high, right gate) were shown in the gate. The numbers indicate the abundance as a percentage (%) of total MAIT cells. [file Image_6.tif]

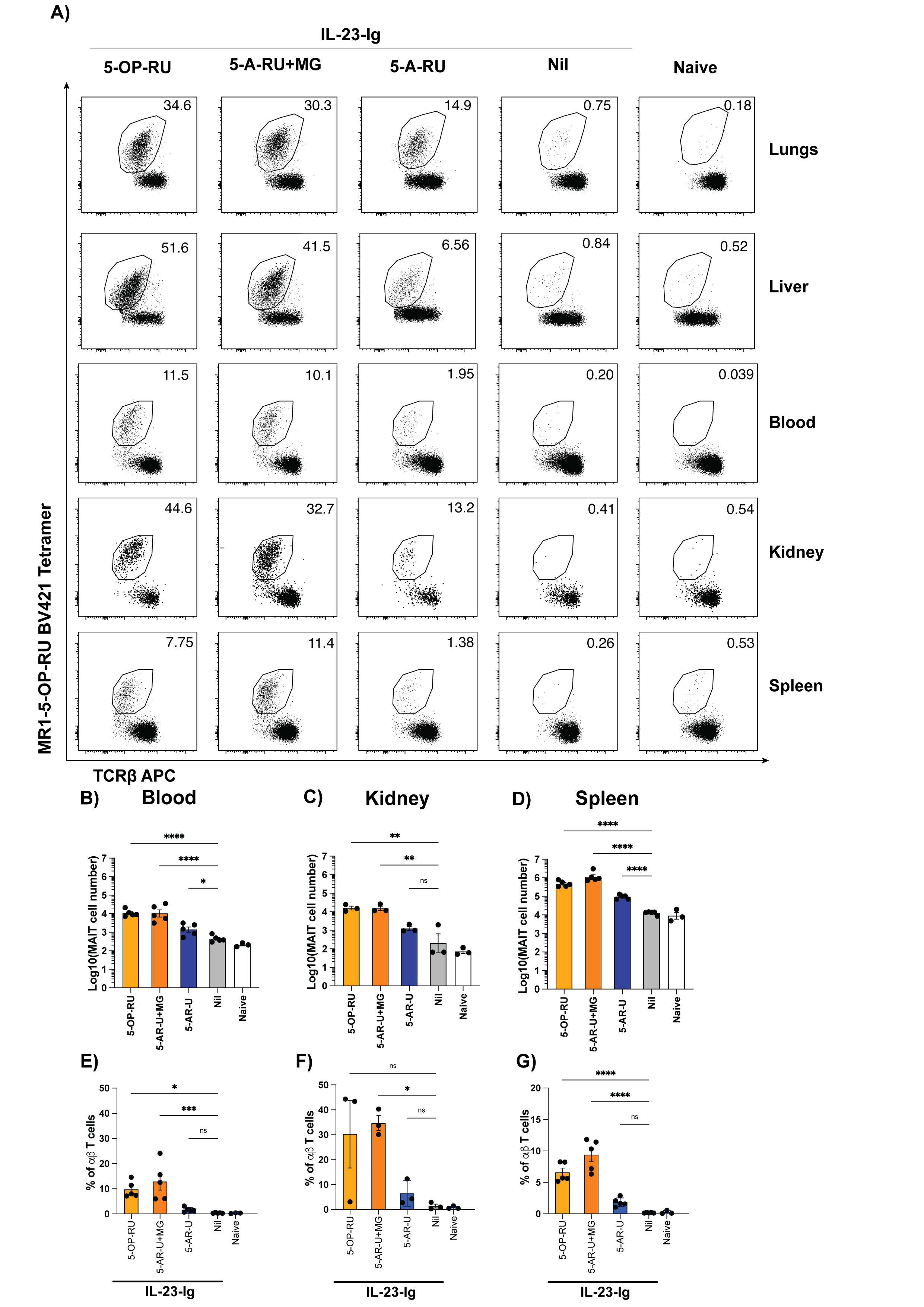

Supplement: Supplementary Figure 7 — (Related to Figure 3 ): MAIT cell enumeration in various tissue from boosting with IL-23-Ig with and without MAIT cell boosting compounds. (A) Representative flow cytometry plots of MAIT cells from the liver, lungs, kidney, spleen, and blood of mice treated with IL-23-Ig plus various compounds and controls. MAIT cells were gated and MAIT cell frequency as a percentage of total αβ T cells shown. Absolute number (B-D) and frequency (E-G) of MAIT cells as a percentage of total αβ T were displayed from indicated groups of mice: Blood: (B, E); Kidney: (C, F); Spleen: (D, G). Data show mean ± SEM and dots represent individual mice (n= 3). Statistical significance is indicated by ns (≥0.05) * (p<0.05), ** (p<0.01), *** (p<0.001); **** (p<0.0001). One-way ANOVA with Tukey correction was performed on log-transformed data or percentage data. [file Image_7.tif]

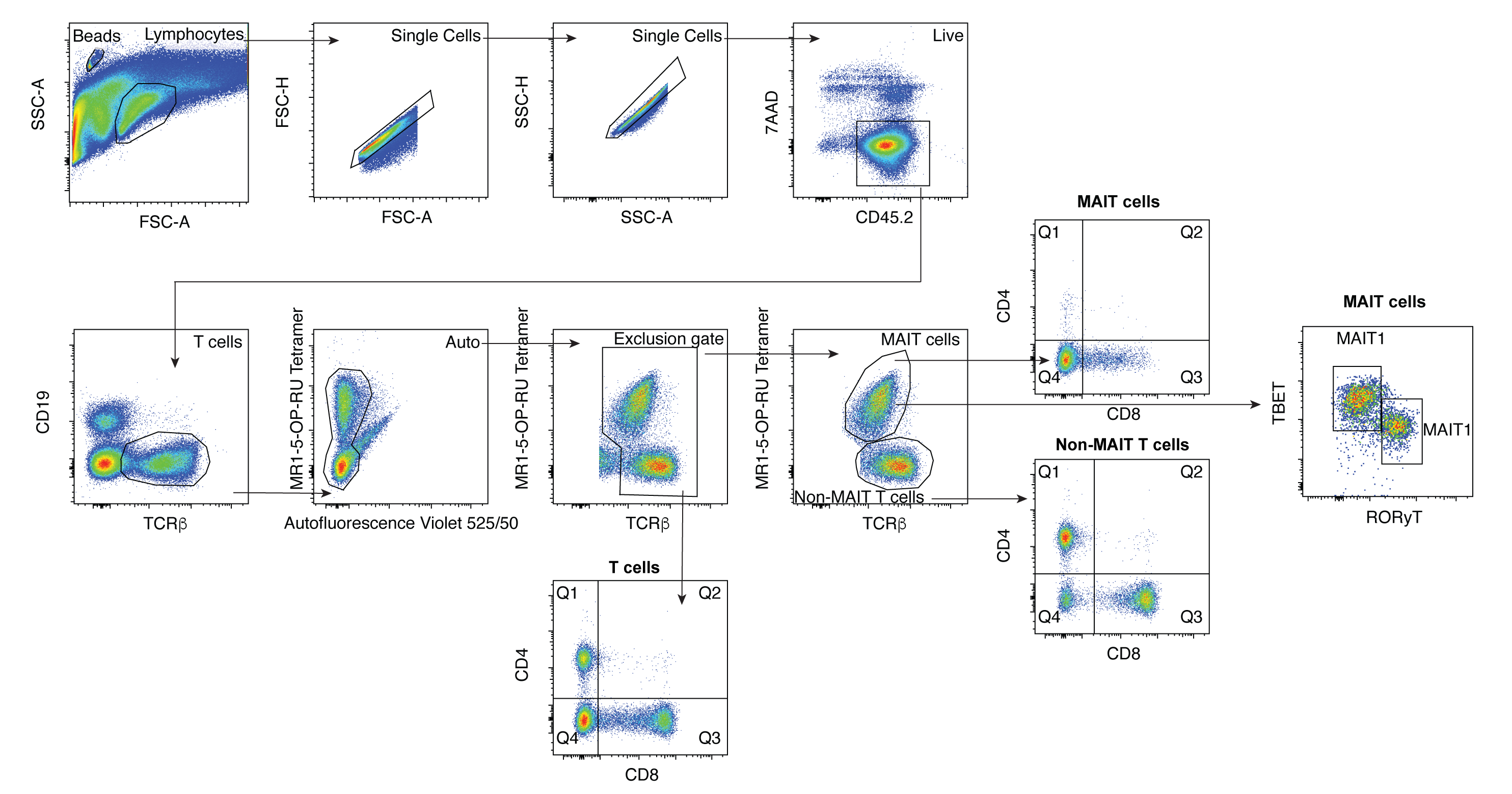

Supplement: Supplementary Figure 8 — (Related to all Figures, except Supplementary Figure 5 ): Cytometric gating strategy for murine MAIT cells. Murine MAIT cells were identified by gating on lymphocytes and then excluding doublets using forward and side scatter (height vs. area). 7-AAD- CD45+ events (live lymphocytes) were gated, and αβ T were identified as TCRβ+CD19-. Auto-fluorescent cells were excluded using violet channel 525/50 (autofluorescence exclusion gate) and any residual non-T cells were excluded based on negative MR1-5-OP-RU tetramer staining (total T cells). Non-MAIT αβ T were gated as TCRβ+ MR1-5-OP-RU tetramer-, and MAIT cells were gated as MR1-5-OP-RU tetramer+ TCRβintermediate cells. Coreceptor expression of CD4 and CD8 was gated on non-MAIT, MAIT and total T cell populations. MAIT1 (T-bet high, RORγT low) and MAIT 17 (T-bet high or low, RORγT high) cells were separated based on T-bet and RORγT expression. This gating strategy was used for all experiments. [file Image_8.tif]

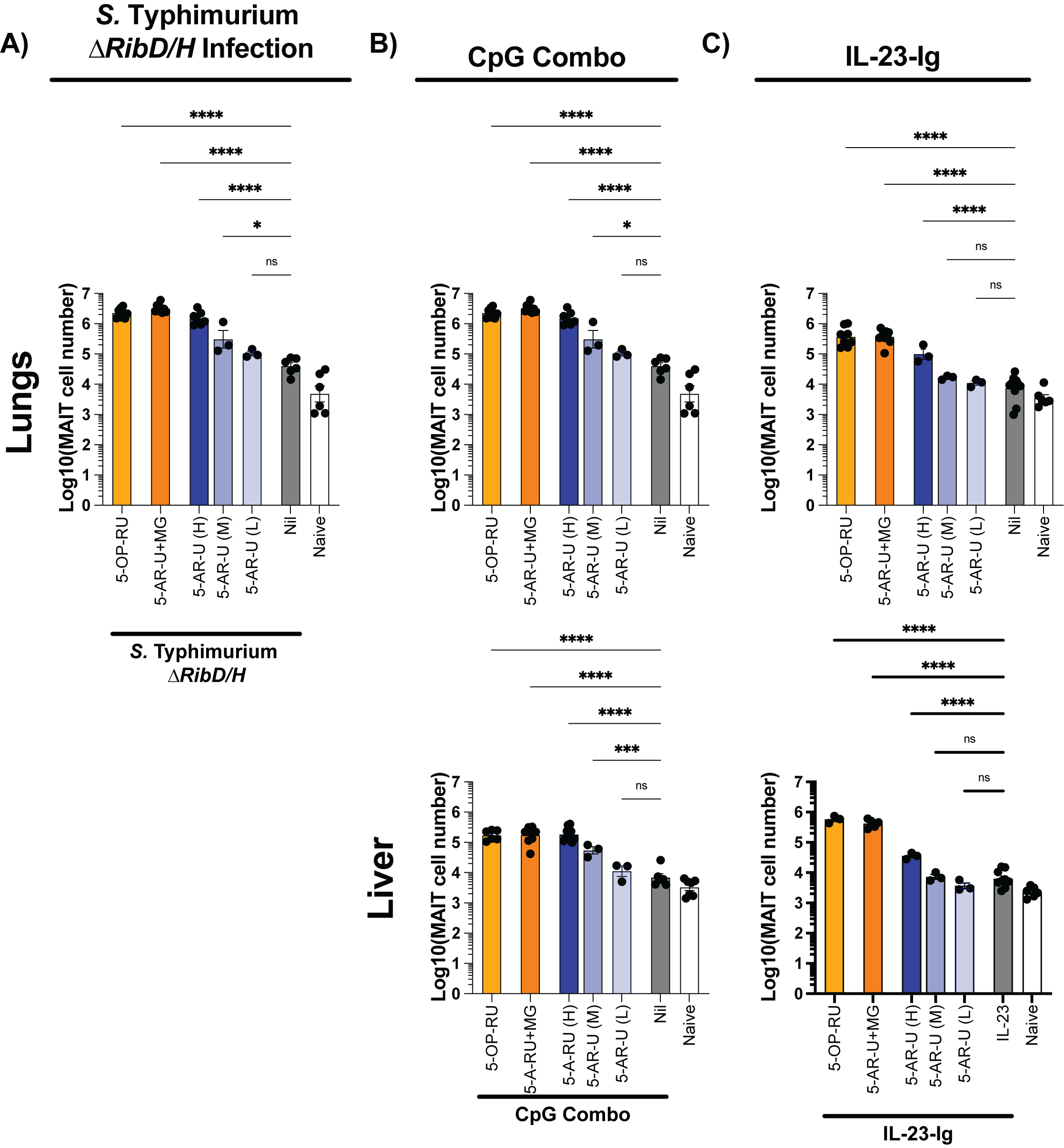

Supplement: Supplementary Figure 9 — Bar graphs showing absolute numbers of MAIT cells in the lungs (top panels) or livers (bottom panels) of (A) Mice infected with 2 x 10^7^ CFU of S. Typhimurium ∆RibD/H and administered four doses (as in Figure 1A ) of either 5-OP-RU (50 pmol, 50 μL), 5-A-RU+MG (5-A-RU+MG: 32.5 nmol +110.5 nmol, 50 μL), a titration of 5-A-RU (5-A-RU (H): 32.5 nmol, 50 μL, 5-A-RU (M): 13 nmol, 50 μL or 5-A-RU (L): 2.6 nmol, 50 μL) or MG (110.5 nmol, 50 μL) IT, or infected with 2.5 x 10^6^ CFU S. Typhimurium BRD509 IT, or naïve mice. (B) CpG combo inoculated mice with four doses (as in Figure 2A ) of either 5-OP-RU (2 nmol, 200 μL), 5-A-RU+MG (5-A-RU+MG: 1.3 μmol +4.42 μmol, 200 μL), a titration of 5-A-RU (5-A-RU (H): 1.3 μmol, 200 μL, 5-A-RU (M): 260 nmol, 200 μL or 5-A-RU (L): 52 nmol, 200 μL), MG (4.42 μmol, 200 μL) or PBS (200 μL) IV or naïve mice. (C) IL-23-Ig plasmid inoculated mice with two doses (as in Figure 3A ) of either 5-OP-RU (200 pmol, 200 μL), 5-A-RU+MG premix (5-A-RU+MG: 130 nmol +442 nmol, 200 μL) IV, a four-dose titration of 5-A-RU (5-A-RU (H): 130 nmol, 200 μL, 5-A-RU (M): 26 nmol, 200 μL or 5-A-RU (L): 5.2 nmol, 200 μL), MG (442 nmol, 200 μL) or naïve mice. Data show mean ± SEM (where n ≥ 3) and dots represent individual mice (n=2, 3 and 5). Statistical analysis was performed on log-transformed data and was performed using a one-way ANOVA with multiple comparisons. *p<0.05. Experiments were performed twice with similar results. [file Image_9.tif]

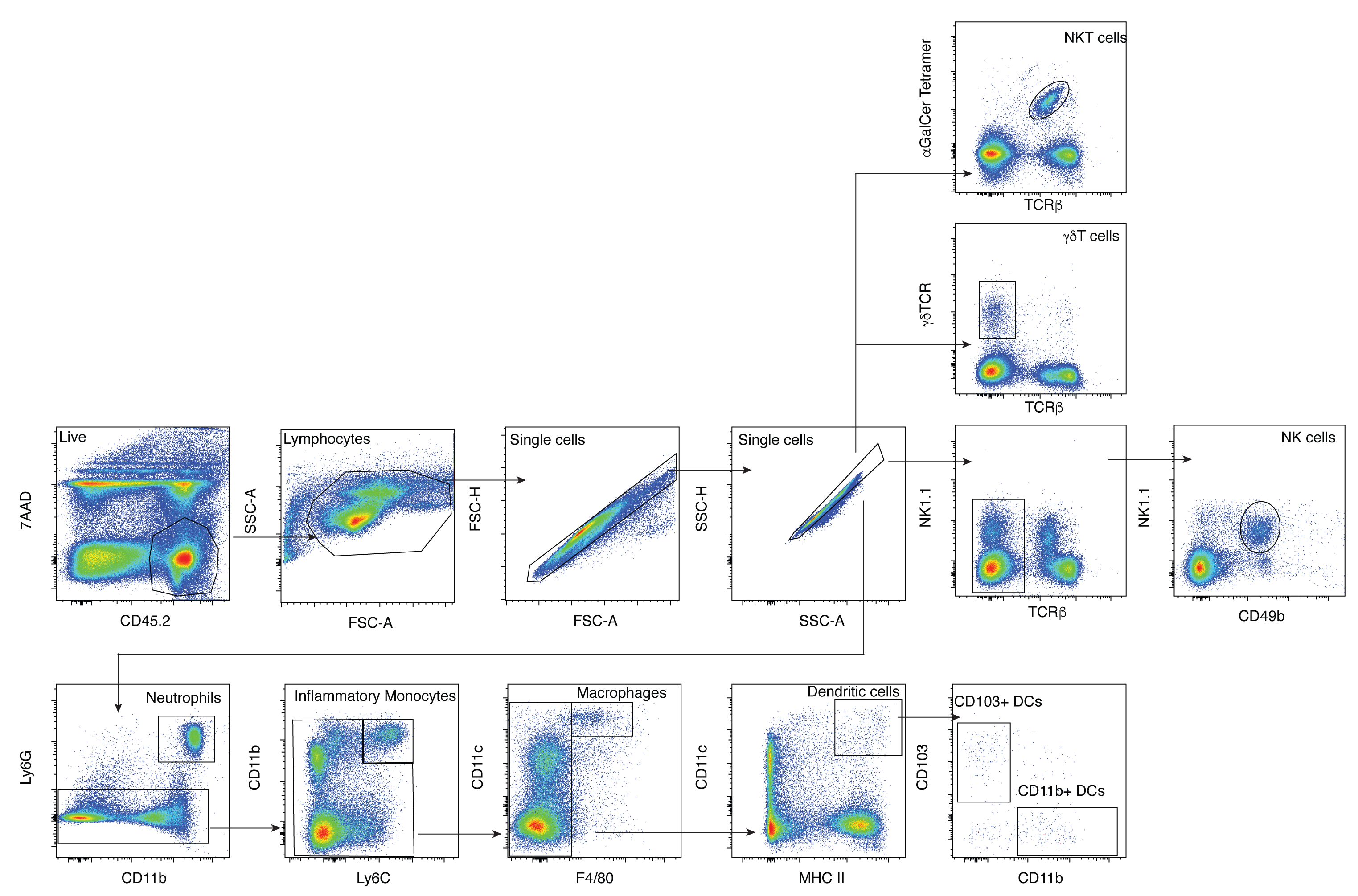

Supplement: Supplementary Figure 10 — (Related to Figures 1 – 3 , Supplementary Figures 2 , 3 ): Cytometric gating strategy for other non-MAIT immune cell subsets. Non-MAIT immune cell subsets were gated similarly as in Supplementary Figure 1 . Live (7AAD-) single immune cells (CD45.2+) were gated out first, then various cell subsets are defined and enumerated with the following cell surface markers. NKT cells (CD1d-αGalCer tetramer+, TCRβ positive), γδ T cells (TCRβ-, γδTCR+), NK cells (TCRβ-, NK1.1+, CD49b), Neutrophils (Ly6G+, CD11b+), Inflammatory monocytes (Ly6G-, Ly6C+, CD11b+), Macrophages (F4/80+, CD11c+), total conventional dendritic cells (DCs) (F4/80-, CD11c+, MHCII+), conventional DC1 (F4/80-, CD11c+, MHCII+, CD103+) conventional cDC2 (F4/80-, CD11c+, MHCII+, CD11b+), were all identified based on the gating strategy above. This strategy was used for all experiments enumerating all non-MAIT and non-T cell subsets. [file Image_10.tif]
